# Supplementary material for: Isolation and characterization of a covalent CeIV-Aryl complex with an anomalous 13C chemical shift
Source: Nat Commun. 2021 Mar 17;12:1713. doi: 10.1038/s41467-021-21766-4 (PMC7969749; doi:10.1038/s41467-021-21766-4)
Supplement: Supplementary file 1 — Supplementary Information [file 41467_2021_21766_MOESM1_ESM.pdf]

Isolation and Characterization of a Covalent Ce^{IV}-Aryl Complex with an Anomalous ^{13}C Chemical Shift

Grace B. Panetti,[a] Dumitru-Claudiu Sergentu,[b] Michael R. Gau,[a] Patrick J. Carroll,[a] Jochen Autschbach,*[b] Patrick J. Walsh,*[a] and Eric J. Schelter*[a]

[a] P. Roy and Diana T. Vagelos Laboratories, Department of Chemistry, University of Pennsylvania, Philadelphia, PA 19104, United States
[b] Department of Chemistry, University at Buffalo State University of New York, Buffalo, NY 14260-3000, (USA)

Phone: EJS (+1)-(215)-898-8633, PJW (+1)-(215)-573-2875; JA (+1)-(716) 645-4122

* e-mail: schelter@sas.upenn.edu, pwalsh@sas.upenn.edu, jochena@buffalo.edu

Supporting Information

Experimental Procedures	2
Synthetic Procedures	5
NMR Data	9
Electrochemical Data	26
Electronic Spectra	30
X-Ray Crystal Structures	31
Additional Computational Data	35
References	48

Experimental Procedures

General Methods. For all reactions and manipulations performed under an inert atmosphere (N_2), standard Schlenk techniques or a Vacuum Atmospheres, Inc. Nexus II drybox equipped with a molecular sieves 13X / Q5 Cu–0226S catalyst purifier system were used. Glassware was oven-dried overnight at 150 °C prior to use. ^{1}H NMR spectra were obtained on a Brüker AM-500 or a Brüker UNI-400 Fourier transform NMR spectrometer at 500 or 400 MHz, respectively. ^{13}C^1 NMR spectra were recorded on a Brüker AM-500 or a Brüker UNI-400 Fourier transform NMR spectrometer at 126 MHz or 100 MHz respectively. ^{19}F NMR spectra were recorded on a Brüker AM-500 or a Brüker UNI-400 Fourier transform NMR spectrometer at 470 MHz or 376 MHz respectively. ^{7}Li NMR spectra were recorded on a Brüker AM-500 or a Brüker UNI-400 Fourier transform NMR spectrometer at 194 MHz or 156 MHz respectively. All spectra were measured at 300 K unless otherwise specified. Chemical shifts were recorded in units of parts per million (ppm) downfield from residual proteo solvent peaks (^{1}H) or characteristic solvent peaks (^{13}C^1). All coupling constants are reported in Hertz.

Solvents.

Tetrahydrofuran, benzene, and pentane were purchased from Fisher Scientific. The solvents were sparged for 20 min with dry N_2 and dried using a commercial two-column solvent purification system comprising columns packed with Q5 reactant and neutral alumina respectively (for benzene and pentane), or two columns of neutral alumina (for THF). Deuterated tetrahydrofuran and benzene were purchased from Cambridge Isotope Laboratories, Inc. both were dried using sodium ketyl and vacuum transferred before use.

Materials.

2,2'-Methylenebis(6-tert-butyl-4-methylphenol) (H_2MBP) was purchased from Sigma-Aldrich and dried for 24 h under dynamic vacuum at 60 °C. Ce(O*t*Bu)$_4$(THF)$_2$ was prepared according to the literature procedure.[2] 4,5-dihydro-4,4-dimethyl-2-[4-(trifluoromethyl)phenyl]-oxazole (H[*ortho*-oxa]) was prepared according to the reported procedure.[3]

Electrochemistry.

Voltammetry experiments (CV, DPV) were performed using a CH Instruments 620D Electrochemical Analyzer/Workstation and the data were processed using CHI software v9.24. All experiments were performed in an N_2 atmosphere drybox using electrochemical cells that consisted of a 4 mL vial, glassy carbon working electrode, a platinum wire counter electrode, and a silver wire plated with AgCl as a quasi-reference electrode. The quasi-reference electrode was prepared by dipping a length of silver wire in concentrated hydrochloric acid.

The working electrode surfaces were polished prior to each set of experiments. Potentials were reported versus ferrocene, which was added as an internal standard for calibration at the end of each run. Solutions employed during these studies were ~3 mM in analyte and 100 mM in [$^{n}Pr_4N$][BAr^F] in 3 mL of tetrahydrofuran. All data were collected in a positive-feedback IR compensation mode.

UV-visible Spectroscopy

10 mm path length quartz cells fused with a J-Young valve were used for UV-vis of air and moisture sensitive compounds. Electronic absorption spectra (UV-Vis) were collected on a Perkin Elmer 950 UV-Vis/NIR spectrophotometer.

X-ray Crystallography

X-ray intensity data were collected on a Brüker APEXII CCD area detector or a Brüker APEXIII D8QUEST CMOS area detector, both employing graphite-monochromated Mo-K α radiation (λ= 0.71073 Å) at 100(1) K. Rotation frames were integrated using SAINT,[4] producing a listing of unaveraged F^2 and $\sigma(F^2)$ values, which were then passed to the SHELXT[5] program package for further processing and structure solution. The intensity data were corrected for Lorentz and polarization effects and for absorption using SADABS[6] or TWINABS.[7] Refinement was performed by full-matrix least squares based on F^2 using SHELXL.[8] All of the reflections were used during refinement. Non-hydrogen atoms were refined anisotropically and hydrogen atoms were refined using a riding model. Structures were rendered using OLEX 2 version 1.3 at 30% probability ellipsoids.[9]

Computational details

Starting from the crystal structure coordinates of **3-THF**, the geometry of **3** was fully optimized for a closed-shell spin-singlet ground-state (GS) with Kohn-Sham density functional theory (DFT), using the 2019 release of the Amsterdam Density Functional (ADF) software package.[10] The popular, all-purposes B3LYP hybrid generalized gradient approximation (GGA) was selected, alongside Slater-type TZ2P basis sets for Ce, N, C, O and F atoms, and the TZP basis set for the H atoms.[11-13] A frozen small-core and good numerical-quality grid settings were applied. Scalar relativistic (SR) effects were treated via the zeroth-order regular approximation (ZORA) all-electron Hamiltonian, as implemented in ADF.[14] Atom-pairwise correction for dispersion forces were accounted for with Grimme's D3 model augmented with the Becke-Johnson (BJ) damping.[15,16] Solvent effects (THF, tetrahydrofuran) on the structure relaxation, and in the NMR calculations, were accounted for with COSMO (conductor-like screening model), a continuum model. The optimized structure compared very well with the crystal structure and was therefore retained for further metal-ligand bond analyses, consisting in natural localized molecular orbital (NLMO) and Bader's quantum theory of atoms-in-molecules (QTAIM) bond analyses.[17,18]

Nuclear magnetic shielding (s) calculations for the C(aryl) atom directly coordinated to the metal center were performed with the NMR module of ADF, using both the scalar relativistic and the spin-orbit, all-electron ZORA Hamiltonians (SR-ZORA, SO-ZORA). These calculations used the relaxed solution geometry, TZ2P basis sets for the Ce, N, C, O and F atoms, the DZP basis set for the H atoms, and various DFT approximations, namely the PBE0 (25% exact exchange) and B3LYP (20%) hybrid GGAs, the PBE (0%) and KT2 (0%) GGAs, and the asymptotically-correct statistical average of orbital potentials (SAOP) model.[1,19-21] The shielding constants were transformed to chemical shifts (δ, ppm) by subtraction from the nuclear magnetic shielding for the C atom in tetramethylsilane (TMS), calculated at the same theoretical level.

Synthetic methods:

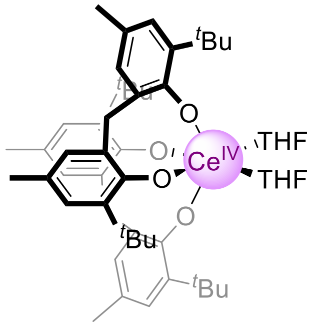

Ce(THF)$_2$(MBP)$_2$ (1): We previously reported a synthesis of **1** that could not be separated from the lithium halide byproducts.[22] This method provides clean **1**. In an N_2 filled drybox, to a clear, colorless solution of H_2MBP (0.270 g, 0.793 mmol, 2 equiv) in 4 mL of THF in a 20 mL scintillation vial with a Teflon coated stir bar, was added a yellow solution of Ce(O^{t}Bu)$_4$(THF)$_2$ (0.200 g, 0.396 mmol, 1 equiv) in a 6 mL solution of 2:1 THF:benzene at room temperature with stirring. The reaction immediately turned an intense purple color and was stirred for 1 hour. The volatile materials were removed under reduced pressure, the residue was triturated with 2 mL of benzene to liberate the *tert*-butanol byproduct, and the volatile materials were again removed under reduced pressure. The resulting purple solid was transferred onto a medium porosity fritted filter and washed with 5 x 2 mL of pentane. The purple solid was dried under reduced pressure for 3 h. Yield: 0.311 g, 0.324 mmol, 82%.

NMR data for this complex was not previously reported and is provided here:
^{1}H NMR (400 MHz, THF-d_8) δ: 7.15 (s, 4H), 6.79 (s, 4H), 5.01 (d, J = 13.4 Hz, 2H) 3.51 (d, J = 14.0 Hz, 2H), 2.31 (s, 12 H), 1.44 (s, 36 H).
^{13}C{^{1}H} NMR (100 MHz, THF-d_8) δ: 168.12, 137.25, 134.45, 129.13, 128.17, 124.12, 35.40, 34.99, 31.15, 20.93.

The quantity of THF present for **1** was verified by ^{1}H-NMR in C_6D_6.

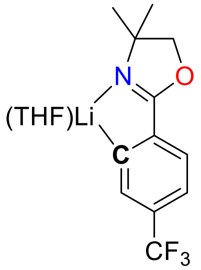

[Li(THF)][*ortho*-oxa] (2): Synthesis adapted from similar compounds.[23] In a N_2 filled drybox, a solution containing H-*ortho*-oxa (1.217 g, 5.0 mmol, 1 equiv) and 10 mL of hexanes in a 20 mL scintillation vial with a Teflon coated stir bar was placed in a −30 °C freezer for 30 mins. The vial was removed from the freezer and, while stirring, a solution of *n*-butyl lithium (2.5 M, 5 mmol, 2 mL) was added dropwise over 5 min. The solution turned from colorless to yellow to brown and a yellow solid precipitated. The reaction mixture was stirred for 50 min at room temperature, after which the solid was collected by filtration over a coarse-porosity fritted-filter and subsequently washed with 3 x 2 mL of hexanes and 1 x 2 mL of pentane. The tan solid was then dried under reduced pressure for 2 h. The solid was then dissolved in minimal THF at rt and then placed in −30 °C freezer overnight. Yellow crystalline blocks formed and were collected over a coarse-porosity fritted-filter and washed with 3 x 2 mL of pentane. The yellow blocks were dried for 2 h under reduced pressure. Yield: 0.831 g, 2.59 mmol, 52 %.

^{1}H NMR (400 MHz, THF-d_8) δ: 8.28 (s, 1H), 7.57 (d, J = 8.0 Hz, 1H), 7.07 (dd, J = 8.0, 2.7 Hz, 1H), 4.17 (s, 2H), 1.34 (s, 6H).
^{13}C{^{1}H} NMR (101 MHz, THF-d_8) δ: 203.98, 172.88, 143.18 (q, J = 2.2 Hz), 137.45 (q, J = 3.3 Hz), 127.47 (q, J = 274.1 Hz), 127.31 (q, J = 28.2 Hz), 124.46, 119.28 (q, J = 4.0 Hz), 80.39, 66.58, 29.03.
^{19}F NMR (376 MHz, THF-d_8) δ: -64.29
^{7}Li NMR (156 MHz, THF-d_8) δ: 2.08
Anal. Cal. for $C_{12}H_{11}F_3LiNO•(C_4H_8O)_{0.5}$: C, 58.96; H, 5.30; N, 4.91. Found C, 59.41; H, 5.41; N, 4.75.

The quantity of THF present for **2** was verified by ^{1}H-NMR in C_6D_6.

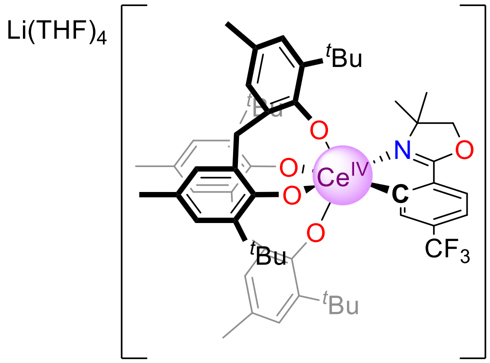

[Li(THF)$_4$][Ce(ortho-oxa)(MBP)$_2$ (3-THF): In an N_2 filled drybox, two 20 mL scintillation vials were placed in a −30 °C freezer. One contained a dark purple solution of **1** (0.200 g, 0.208 mmol, 1 equiv) in 4 mL of benzene with a Teflon coated stir bar and the other contained a yellow solution of **2** (0.067 g, 0.208 mmol, 1 equiv) in 4 mL of benzene. After cooling for 30 min, the now frozen solution of **2** was removed from the freezer and allowed to thaw. Immediately upon thawing, the frozen solution of **1** was removed from the freezer and the solution of **2** was added dropwise at rt over 2 min. Upon mixing, the solution immediately changed from a dark purple to a dark red color and was allowed to stir for 5 min at rt. At this point the volatile materials were removed under vacuum. The resulting solid was redissolved in a mixture of 3 mL of toluene and 8 drops of THF in an 8 mL scintillation vial. This solution was layered with 5 mL of pentane and placed in a −30 °C freezer for 3 days. During this time, red crystals formed, and were collected by filtration over a medium porosity fritted filter and washed with cold pentane 5 x 2 mL. Yield: 0.198 g, 0.137 mmol, 66 %.

^{1}H NMR (500 MHz, THF-d_8) δ: 8.53 (s, 1H), 7.78 (d, J = 8.0 Hz, 1H), 7.12 (d, J = 7.5 Hz, 1H), 7.01 (s, 2H), 6.92 (s, 2H), 6.75 (s, 1H), 6.66 (s, 1H), 6.62 (s, 1H), 6.58 (s, 1H), 5.10 (d, J = 13.4 Hz, 1H), 4.72 (d, J = 13.5 Hz, 1H), 4.32 (s, 1H), 4.01 (s, 1H), 3.23 (d, J = 13.4 Hz, 1H), 3.10 (d, J = 13.6 Hz, 1H), 2.27 – 2.05 (m, 12H), 1.57 (s, 3H), 1.45 (s, 9H), 1.39 (s, 9H), 1.24 (s, 3H), 1.16 (s, 9H), 1.08 (s, 9H).
^{13}C{^{1}H} NMR (126 MHz, THF-d_8) δ: 255.58, 174.49, 168.21, 167.40, 166.81, 137.97, 137.72, 137.33, 137.11, 136.98, 136.79, 135.03, 134.90, 134.58, 132.58 (q, J = 3.1 Hz), 131.11 (q, J = 29.3 Hz), 128.85, 128.75, 128.61, 127.02, 126.64 (q, J = 177 Hz), 126.08, 124.25, 124.11, 123.95, 123.60,

120.18 (q, J = 4.1 Hz), 82.35, 68.88, 35.89, 35.71, 35.60, 35.03, 32.46, 31.54, 31.09, 30.97, 30.16, 21.31, 21.17.
^{19}F NMR (470 MHz, THF-d_8) δ: -62.44
^{7}Li NMR (194 MHz, THF-d_8) δ: -0.57
X-ray quality crystals were obtained from a vapor diffusion of pentane into concentrated solutions of **3-THF** in a solution consisting of 1:2 THF:toluene in a –30° C freezer.
Anal. Cal. for $C_{74}H_{99}CeF_3LiNO_9$•(C_7H_8): C, 65.61; H, 7.66; F, 3.95; N, 1.03. Found C, 65.21; H, 6.65; N, 1.30. Best result of 3 attempts.
UV-Vis: λ=460 nm (ε= 7,533 $Lmol^{-1}cm^{-1}$), λ=292 nm (ε= 24,426 $Lmol^{-1}cm^{-1}$).

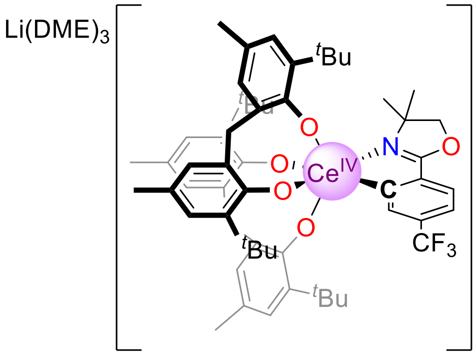

[Li(DME)$_3$][Ce(ortho-oxa)(MBP)$_2$ (3-DME): In an N_2 filled drybox, two 20 mL scintillation vials were placed in a −30 °C freezer. One contained a dark purple solution of **1** (0.100 g, 0.104 mmol, 1 equiv) in 2 mL of benzene with a Teflon coated stir bar and the other contained a yellow solution of **2** (0.034 g, 0.104 mmol, 1 equiv) in 2 mL of benzene. After cooling for 30 min, the now frozen solution of **2** was removed from the freezer and allowed to thaw. Immediately upon thawing, the frozen solution of **1** was removed from the freezer and the solution of **2** was added dropwise at rt over 2 min. Upon mixing, the solution immediately changed from a dark purple to a dark red color and was allowed to stir for 5 min at rt. At this point the volatile materials were removed under reduced pressure. The resulting solid was dissolved in of 3 mL of DME in an 8 mL scintillation vial. This solution was layered with 5 mL of pentane and placed in a −30 °C freezer for 3 days. During this time, red crystals formed, and were collected by filtration over a medium porosity fritted filter and washed with cold pentane 5 x 2 mL. Yield: 0.111 g, 0.079 mmol, 75 %.

^{1}H NMR (500 MHz, THF-d_8) δ: 8.53 (s, 1H), 7.80 (d, J = 7.9 Hz, 1H), 7.13 (dd, J = 8.1 Hz, 2.6 Hz, 1H), 7.01 (s, 2H), 6.91 (s, 2H), 6.76 (s, 1H), 6.68 (s, 1H), 6.64 (s, 1H), 6.59 (s, 1H), 5.12 (d, J = 13.3 Hz, 1H), 4.72 (d, J = 13.4 Hz, 1H), 4.33 (d, J = 7.6 Hz, 1H), 4.01 (d, J = 6.6 Hz 1H), 3.24 (d, J = 13.6 Hz, 1H), 3.11 (d, J = 13.6 Hz, 1H), 2.24 (s, 3H), 2.19 (s, 9H), 1.58 (s, 3H), 1.46 (s, 9H), 1.40 (s, 9H), 1.25 (s, 3H), 1.17 (s, 9H), 1.09 (s, 9H).
^{13}C{^{1}H} NMR (126 MHz, THF-d_8) δ: 255.61, 174.55, 168.23, 167.46, 166.87, 138.03, 137.77, 137.40, 137.18, 137.05, 136.84, 135.08, 134.94, 134.63, 132.65 (q, J = 3.5 Hz), 131.18 (q, J = 29.5 Hz), 129.03, 128.82, 128.66, 127.22 (q, J = 177 Hz), 127.06, 126.14, 124.29, 124.18, 123.99, 123.66, 120.24 (q, J = 4.0 Hz), 82.41, 68.93, 35.94, 35.76, 35.68, 35.09, 32.52, 31.59, 31.14, 31.03, 30.22, 21.36, 21.21.

^{19}F NMR (470 MHz, THF-d_8) δ: -62.44
^{7}Li NMR (194 MHz, THF-d_8) δ: -0.57
X-ray quality crystals were obtained from a layering of pentane on top of a saturated solution of 3-DME in DME (1:1, DME:Pentane).
Anal. Cal. for $C_{70}H_{101}CeF_3LiNO_{11}$: C, 62.90; H, 7.62; N, 1.05. Found C, 62.45; H, 7.32; N, 1.55.

NMR Data

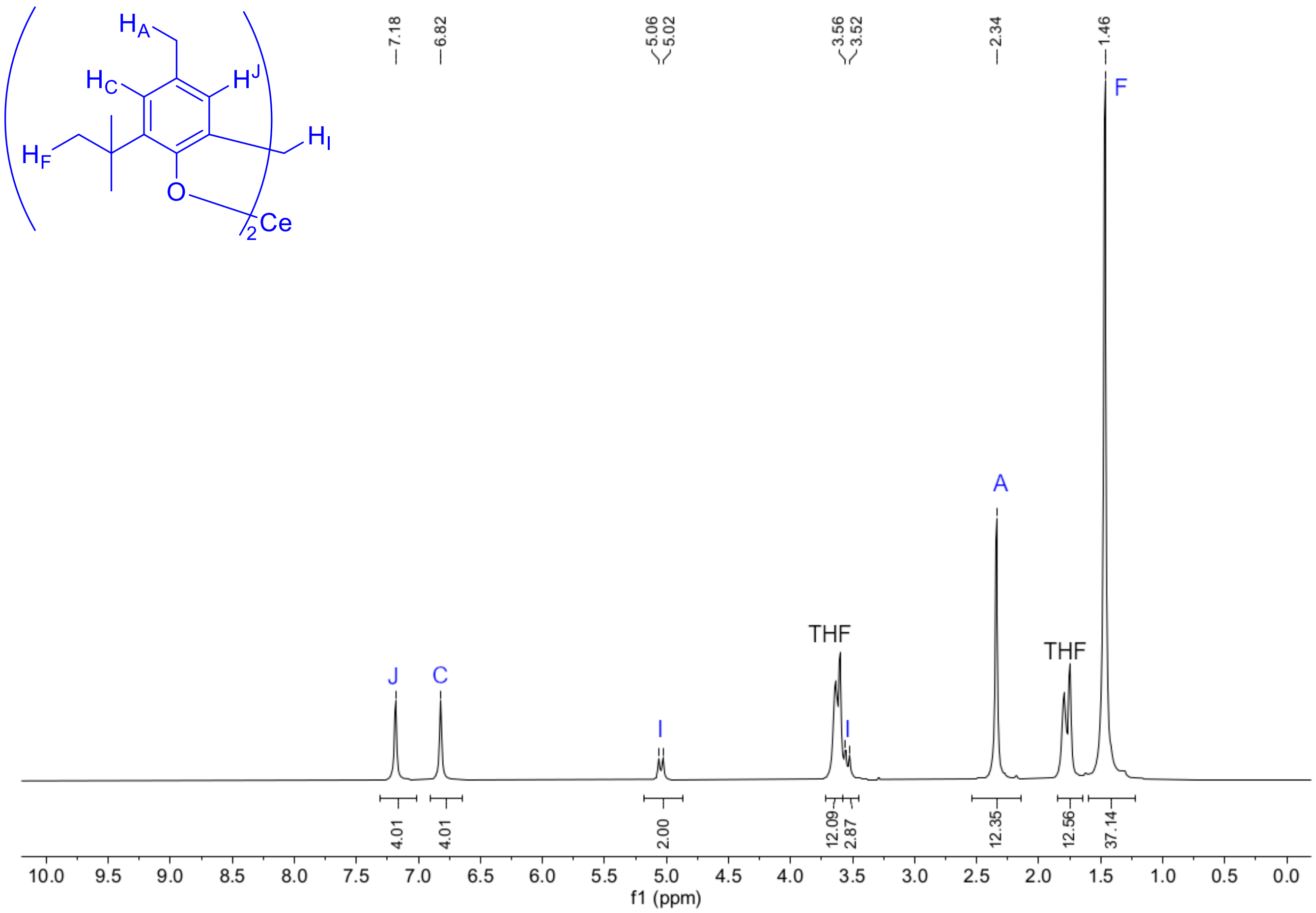

Supplementary Figure 1: ^{1}H NMR (400 MHz, THF-d_8) spectrum of **1.**

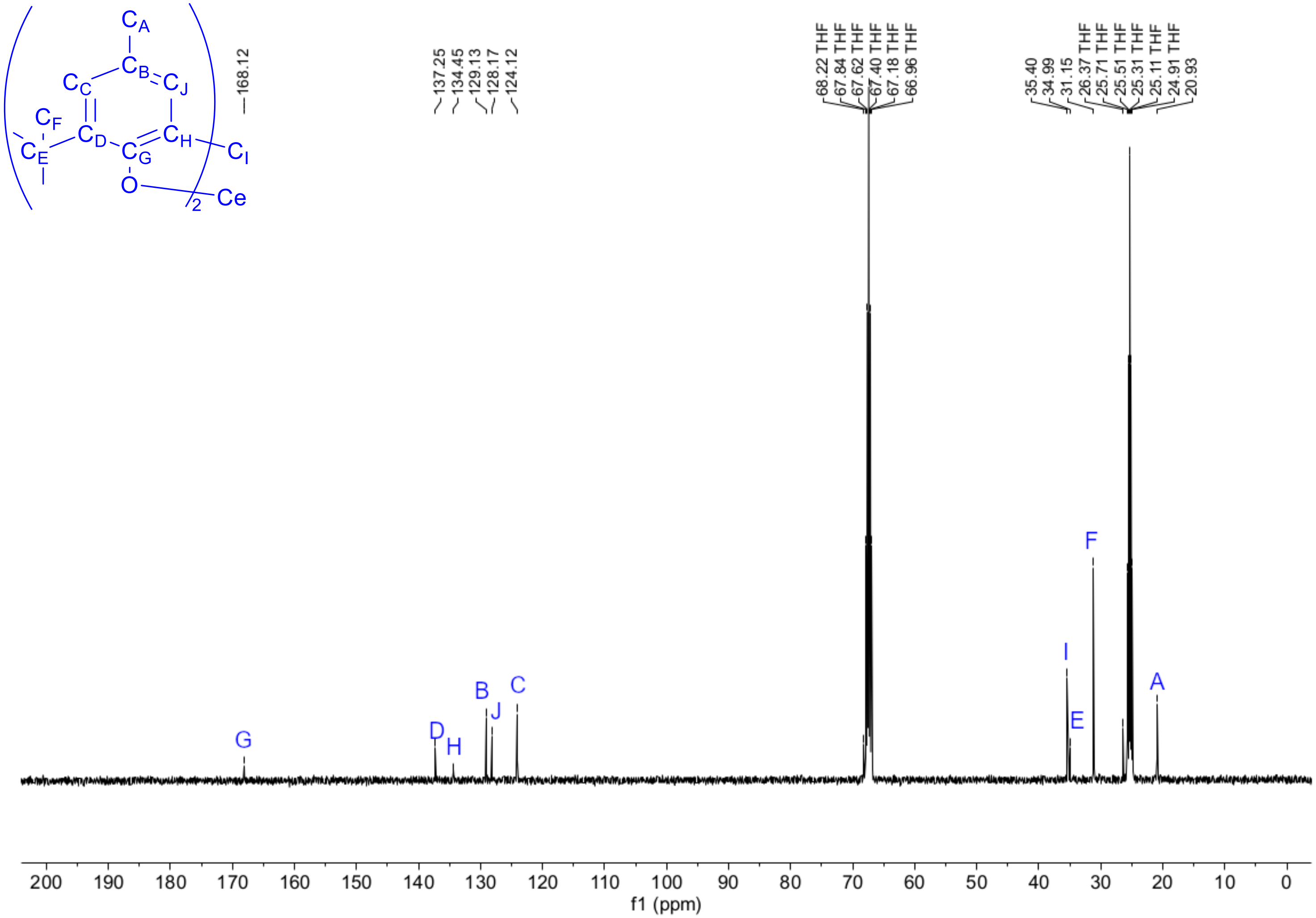

Supplementary Figure 2: $^{13}C\{^1H\}$ NMR (101 MHz, THF-d_8) spectrum of **1.**

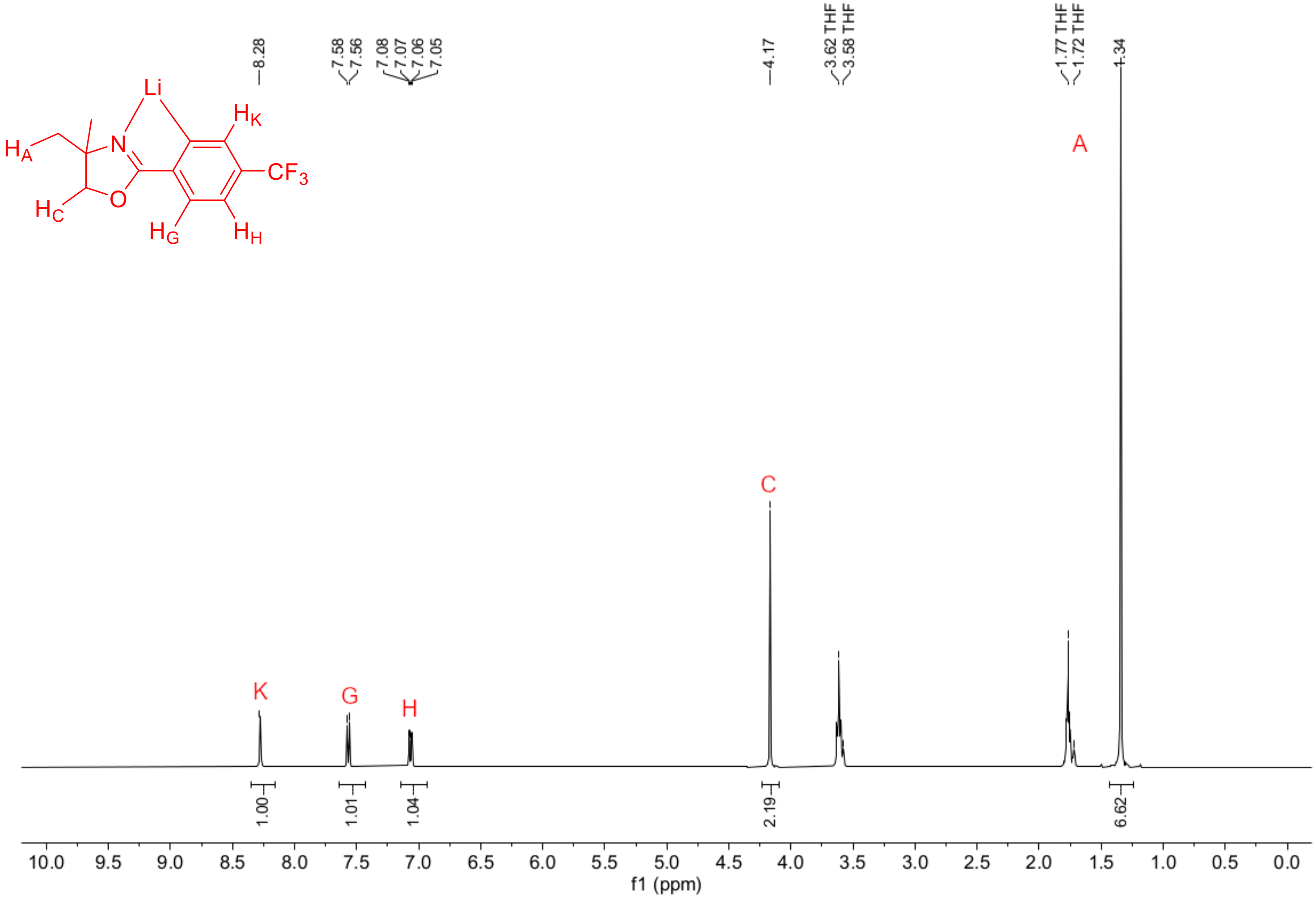

Supplementary Figure 3: ^{1}H NMR (400 MHz, THF-d_8) spectrum of **2.**

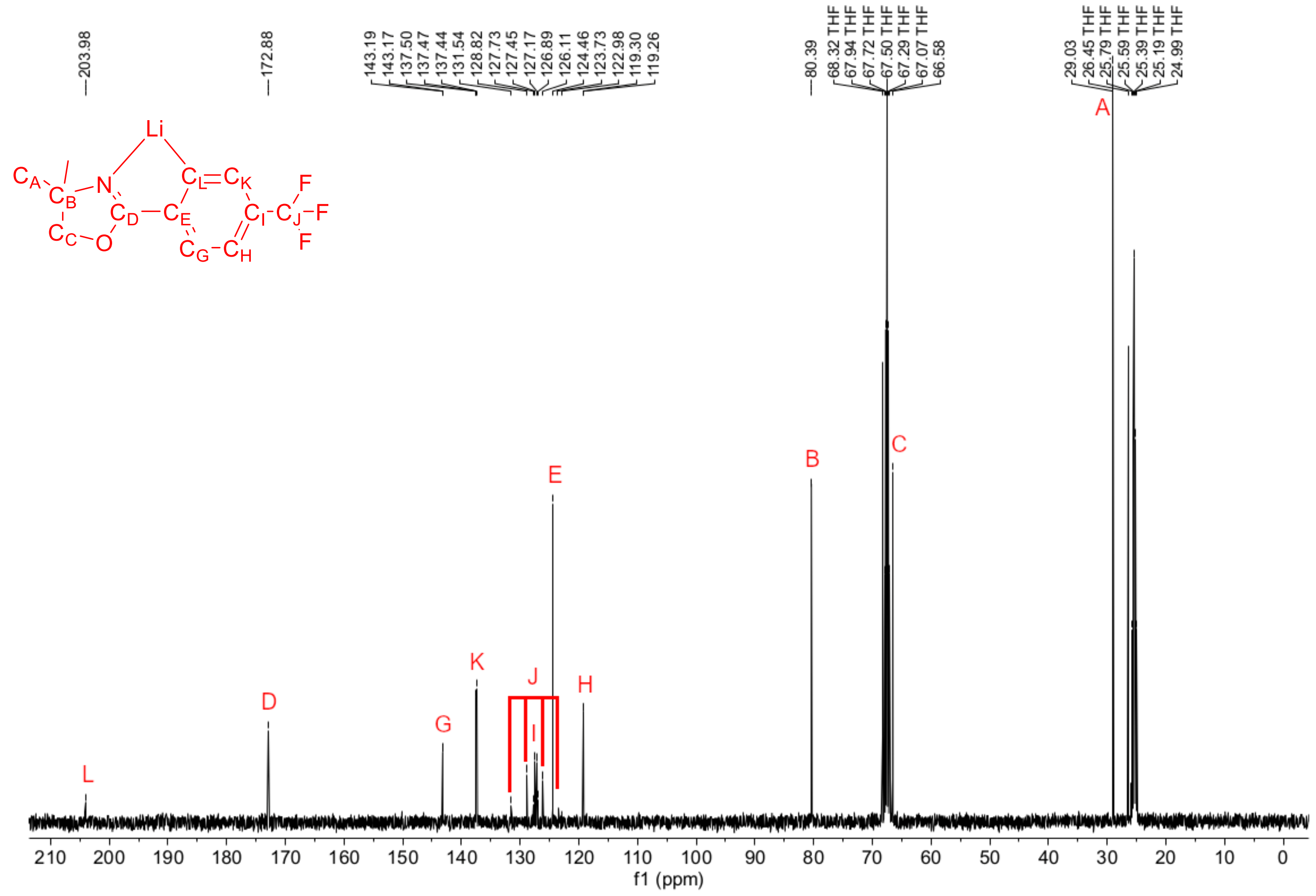

Supplementary Figure 4: $^{13}C\{^1H\}$ NMR (101 MHz, THF-d_8) spectrum of **2.**

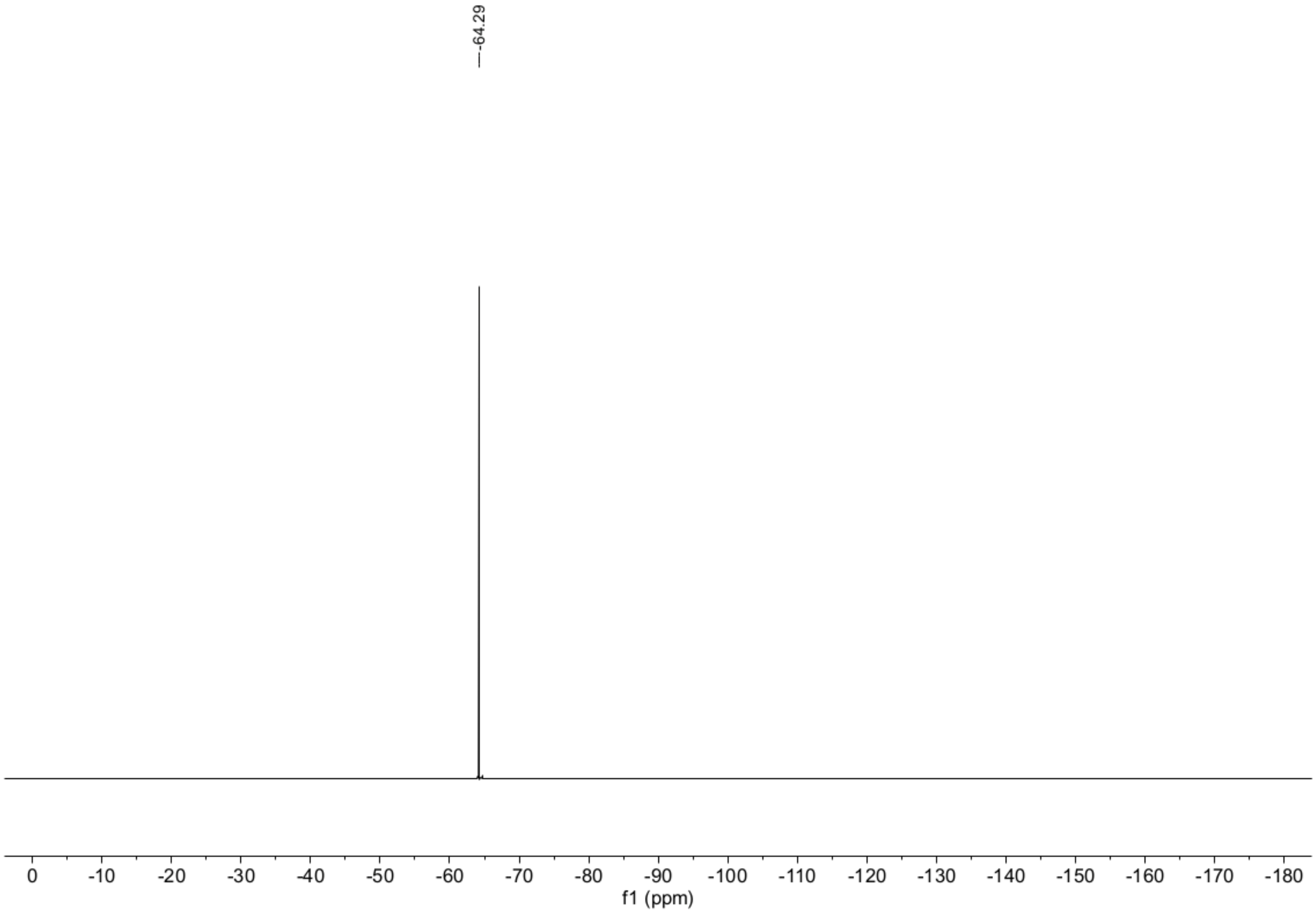

Supplementary Figure 5: ^{19}F NMR (376 MHz, THF-d_8) spectrum of **2.**

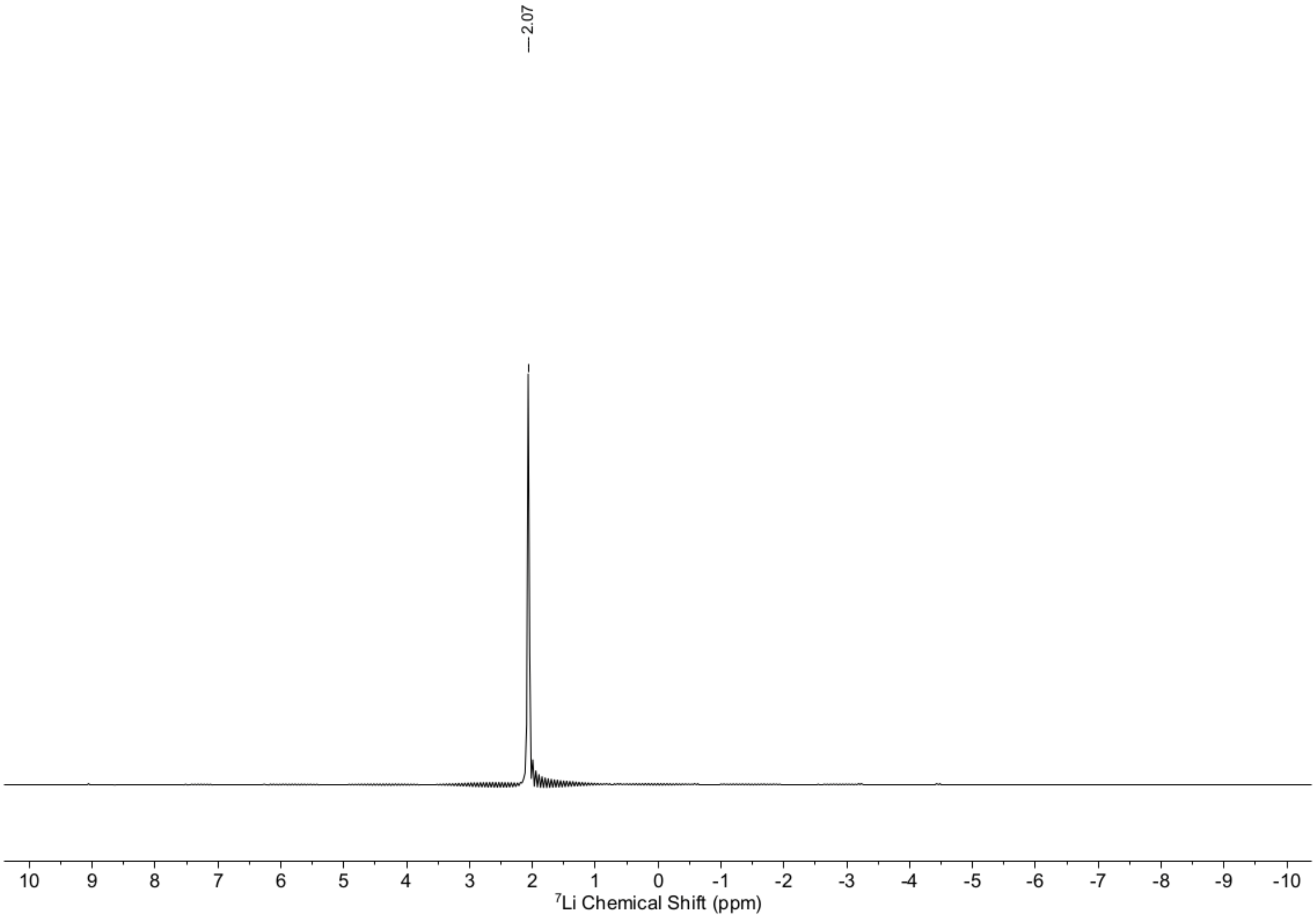

Supplementary Figure 6: ^{7}Li NMR (156 MHz, THF-d_8) spectrum of **2.**

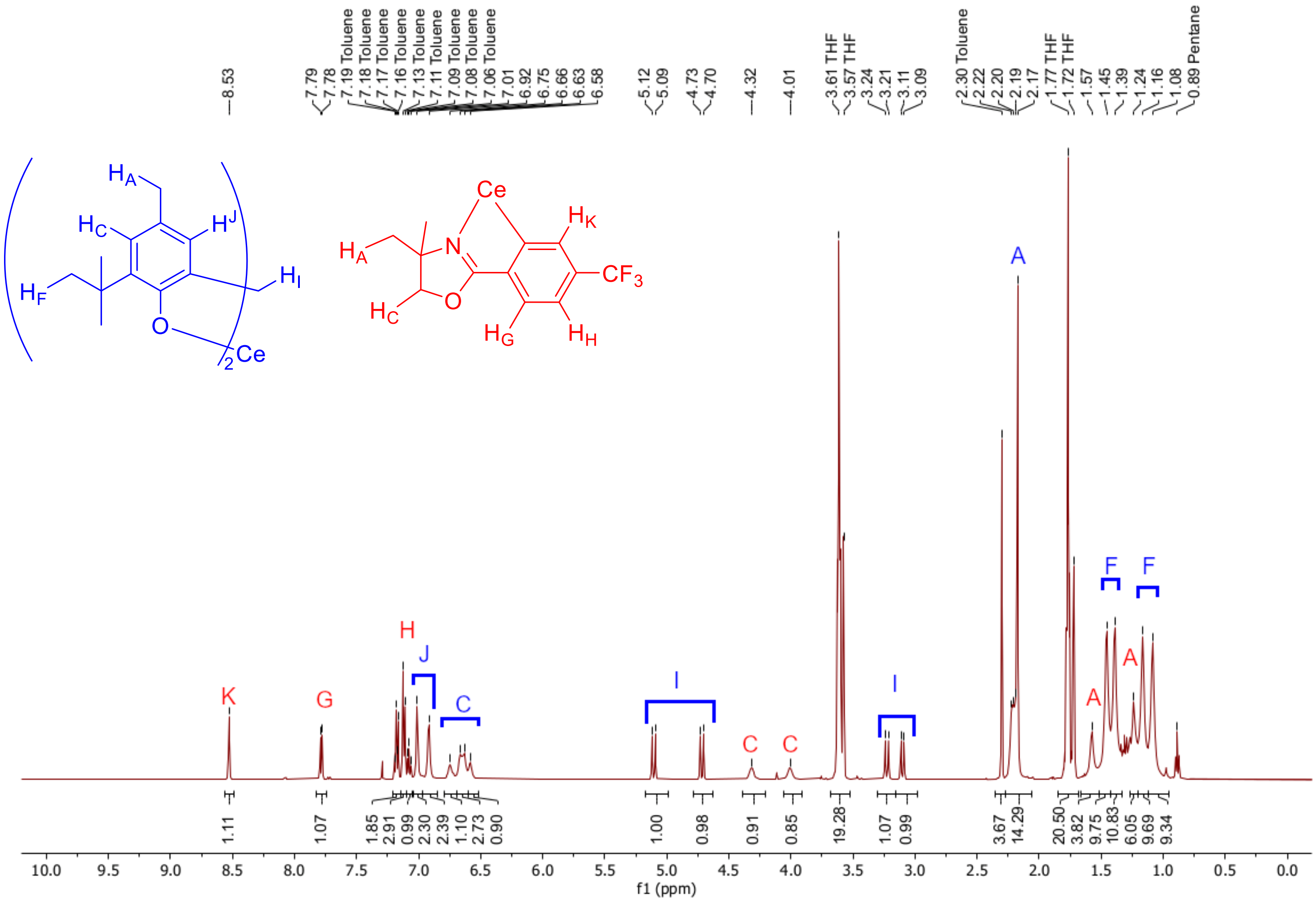

Supplementary Figure 7: ^{1}H NMR (500 MHz, THF-d_8) spectrum of **3-THF.**

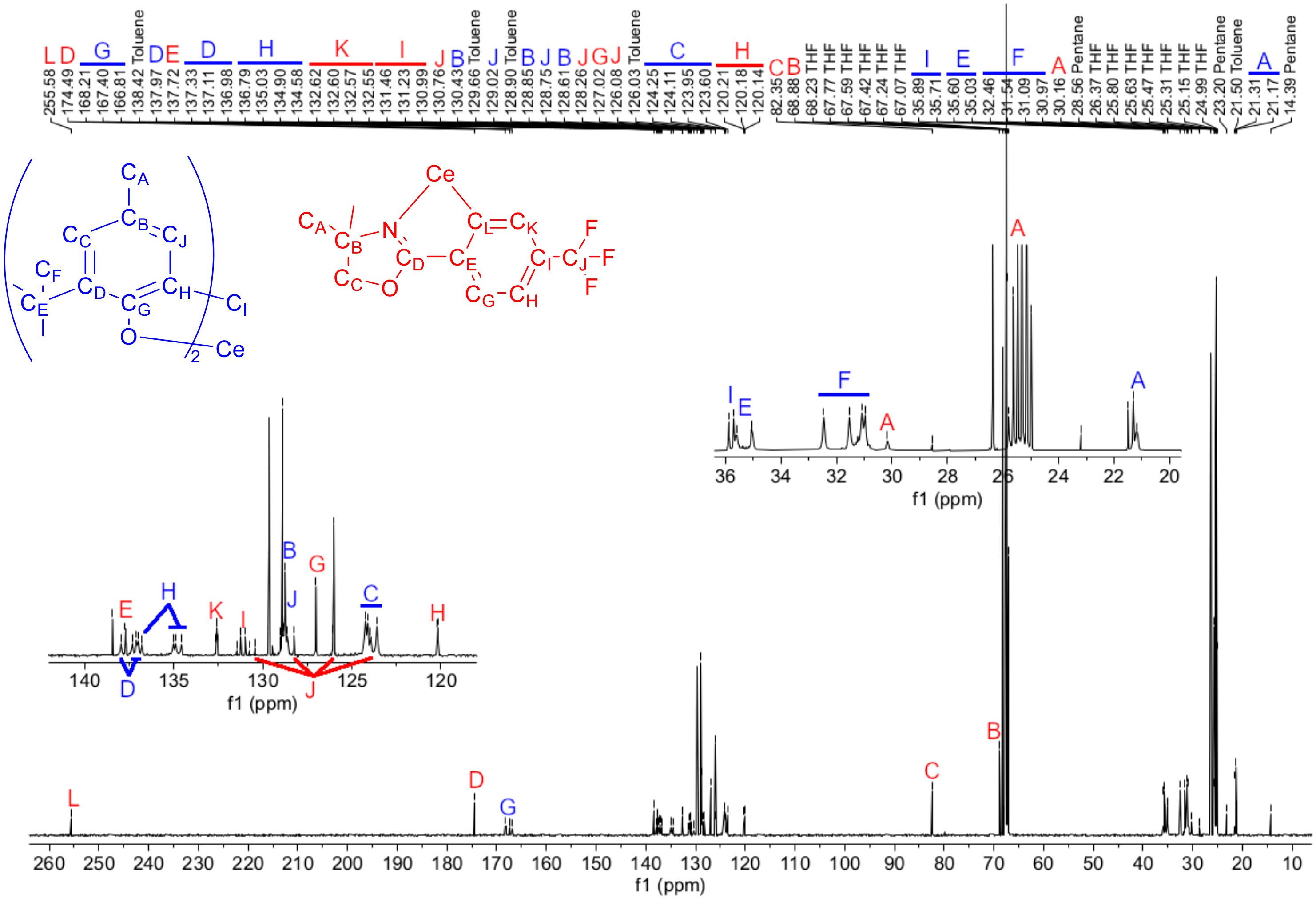

Supplementary Figure 8: $^{13}C\{^{1}H\}$ NMR (126 MHz, THF-d_8) spectrum of **3-THF.**

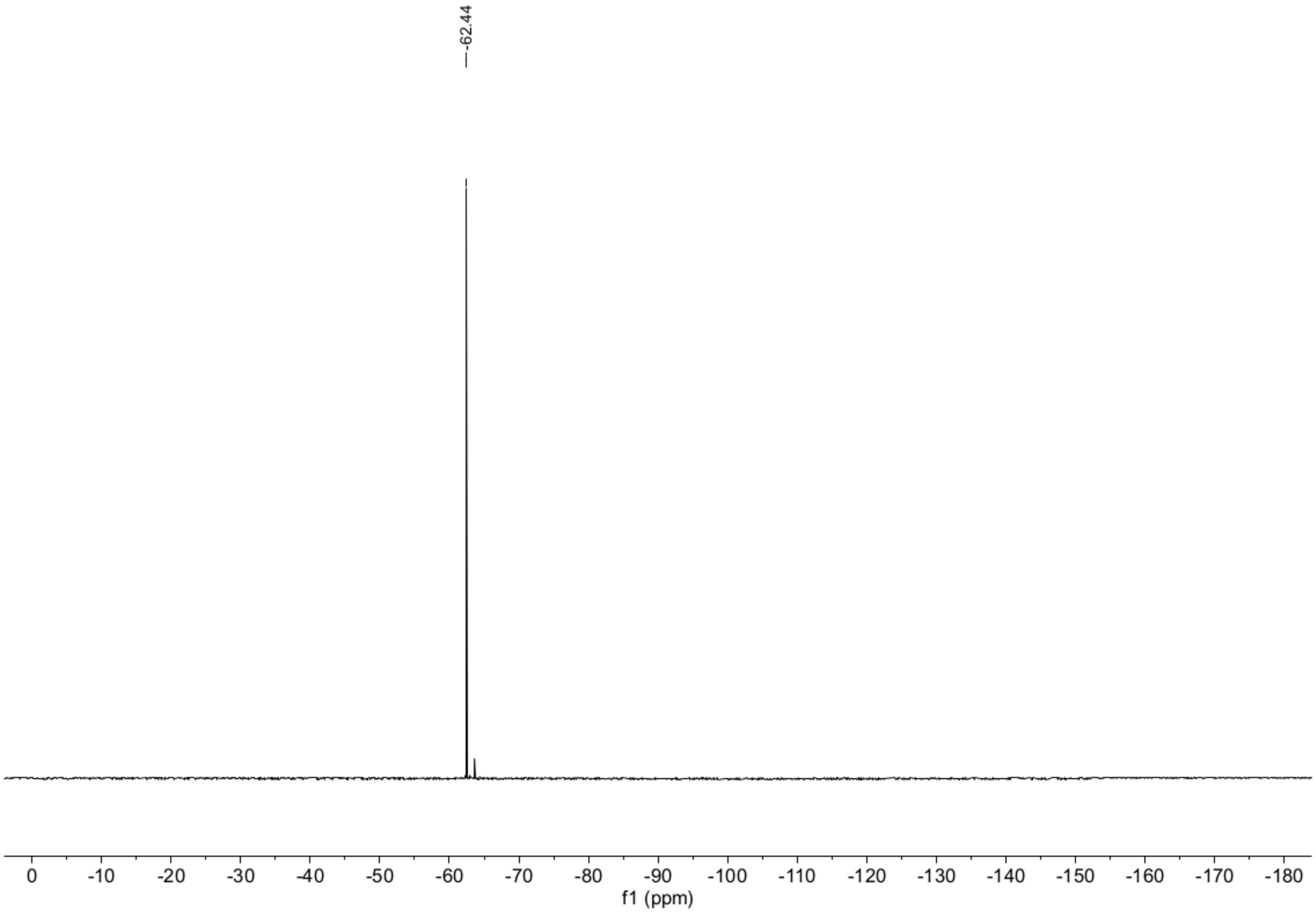

Supplementary Figure 9: ^{19}F NMR (470 MHz, THF-d_8) spectrum of **3-THF.**

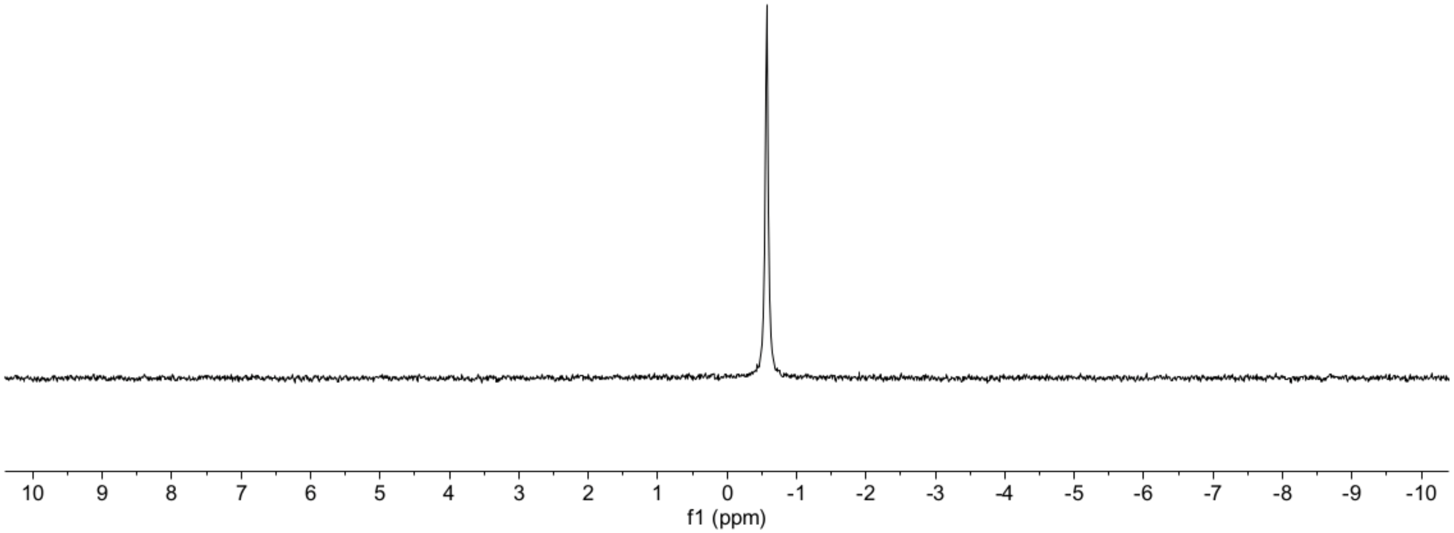

Supplementary Figure 10: ^{7}Li NMR (194 MHz, THF-d_8) spectrum of **3-THF.**

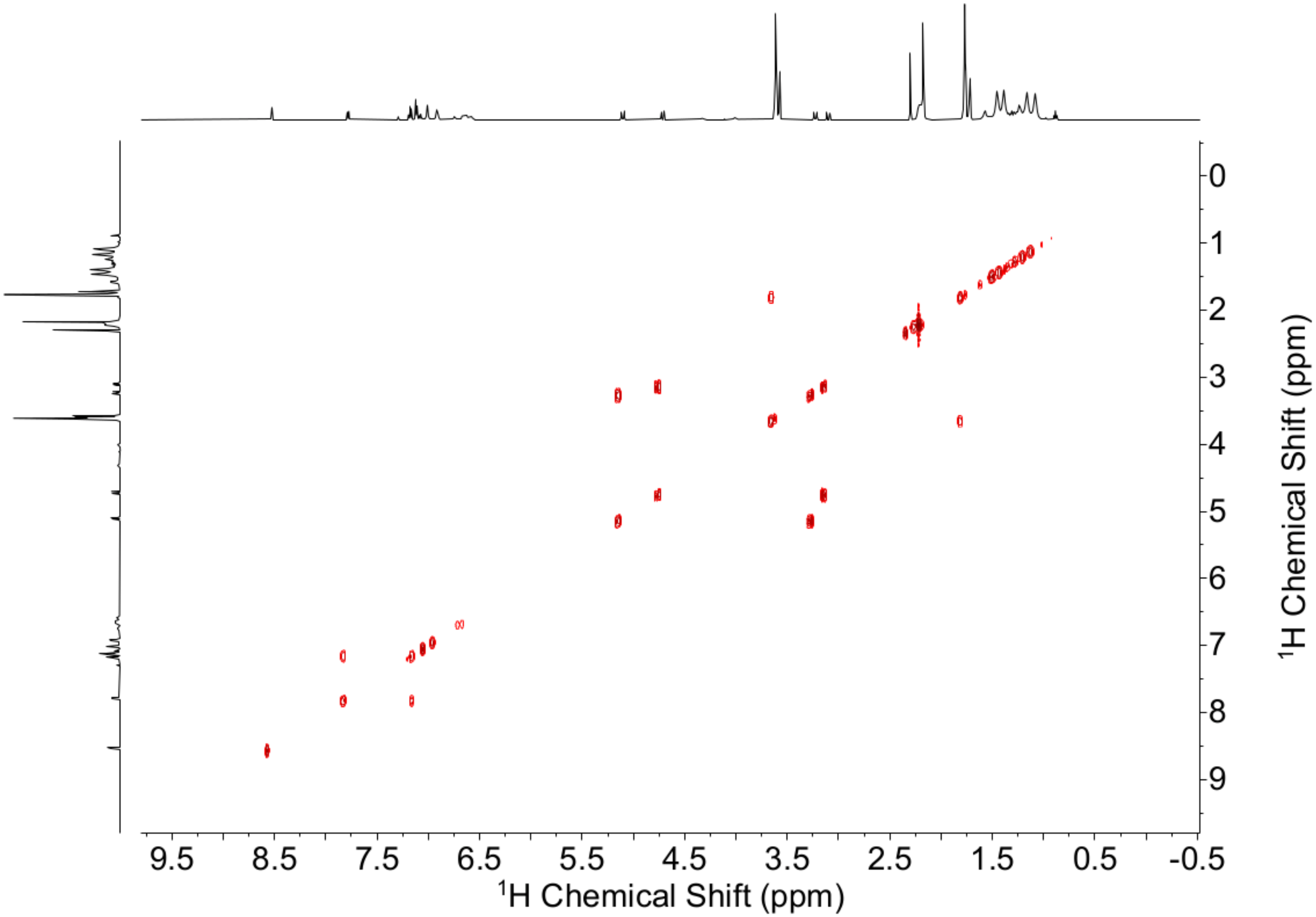

Supplementary Figure 11: COSY spectrum of **3-THF** recorded in THF-d_8.

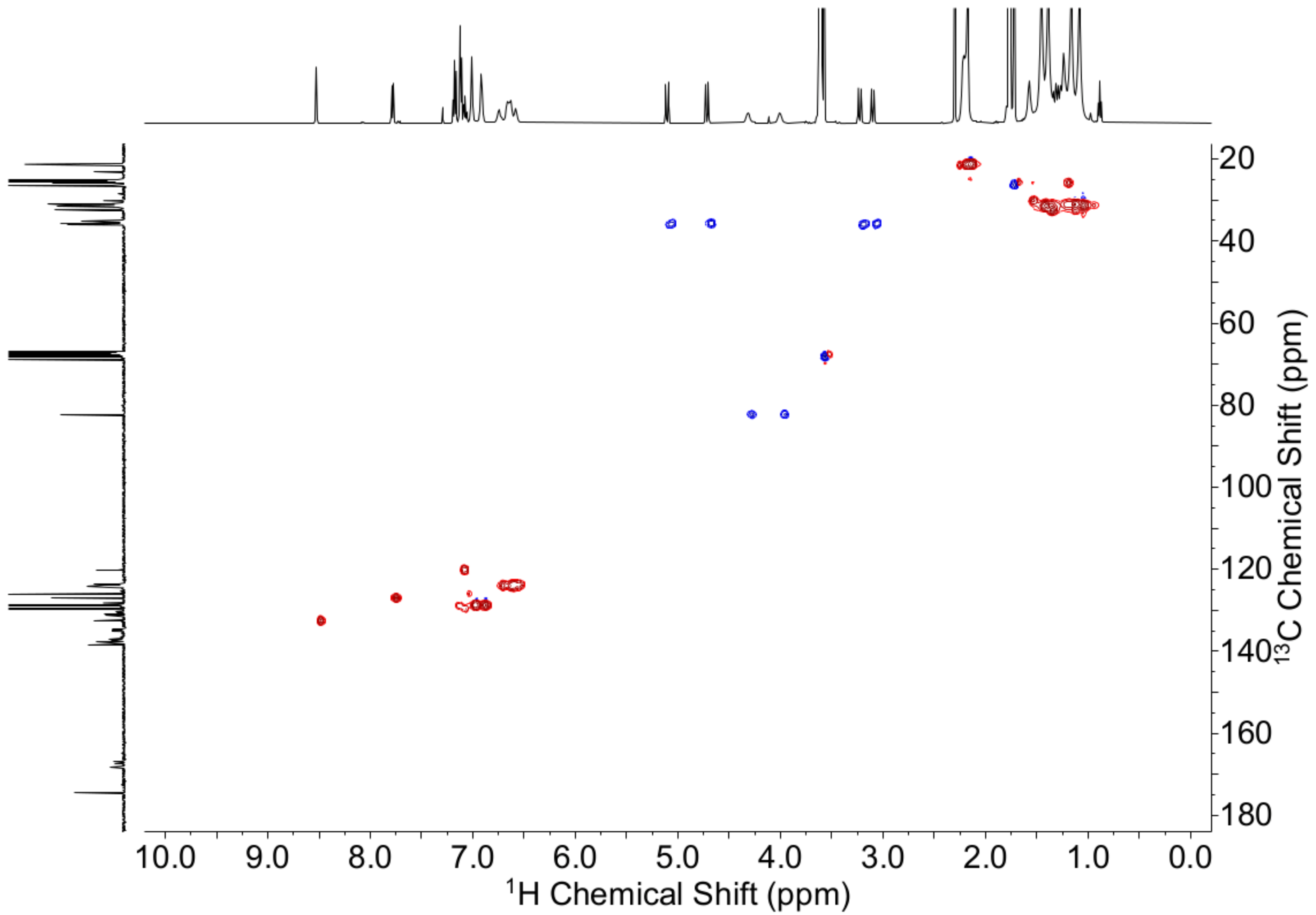

Supplementary Figure 12: HSQC spectrum of **3-THF** recorded in THF-d_8.

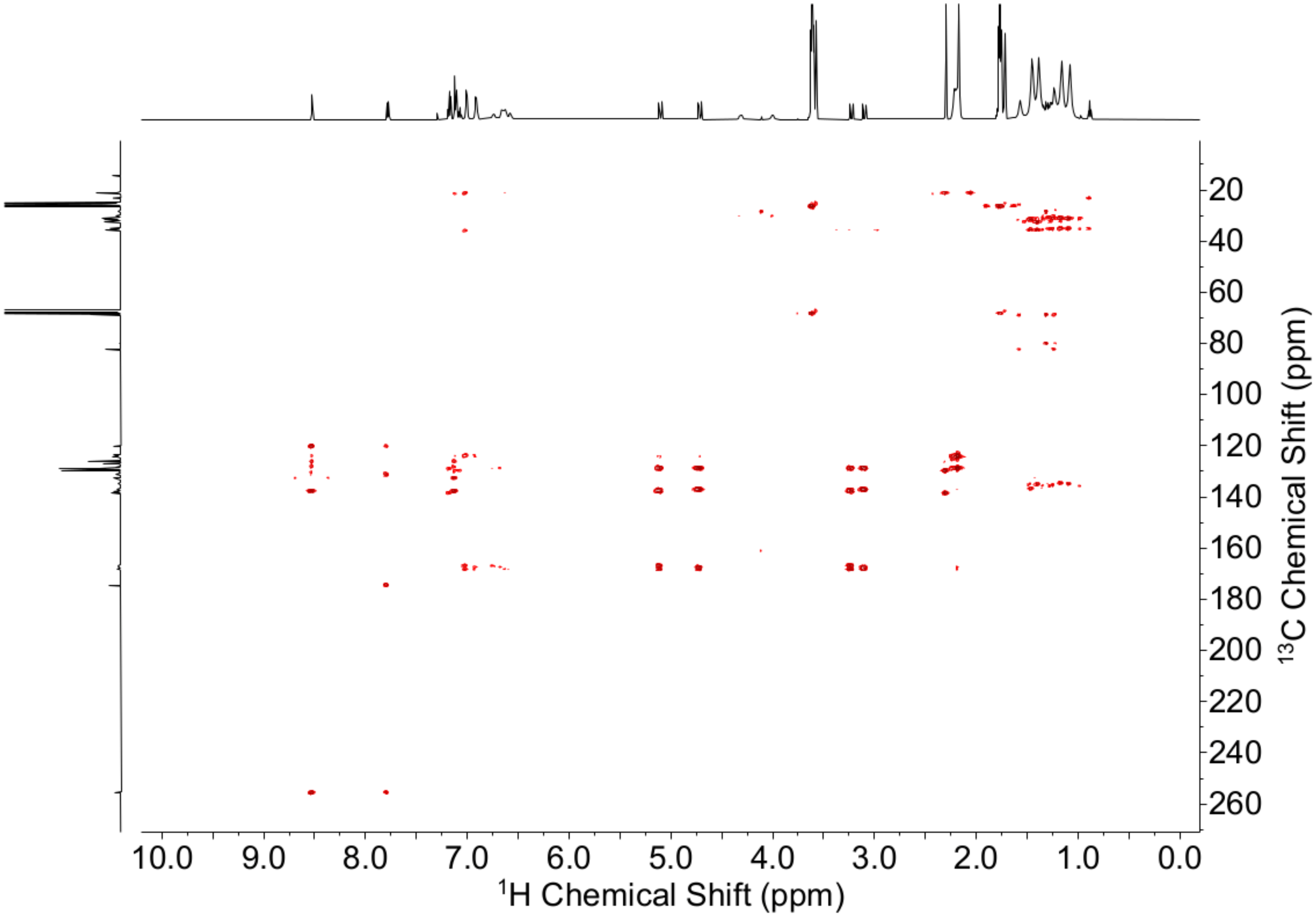

Supplementary Figure 13: HMBC spectrum of **3-THF** recorded in THF-d_8.

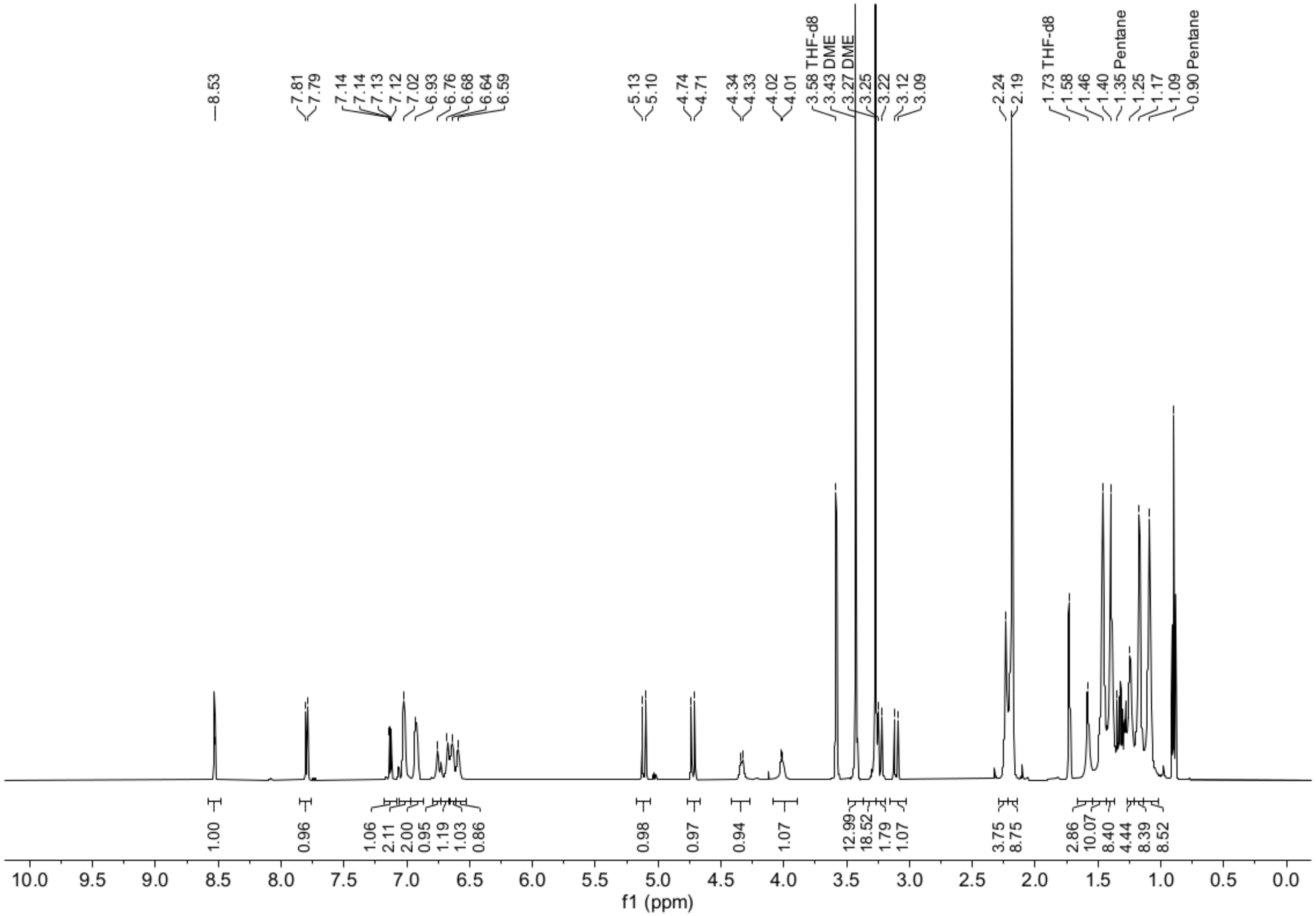

Supplementary Figure 14: ^{1}H NMR (500 MHz, THF-d_8) spectrum of **3-DME.**

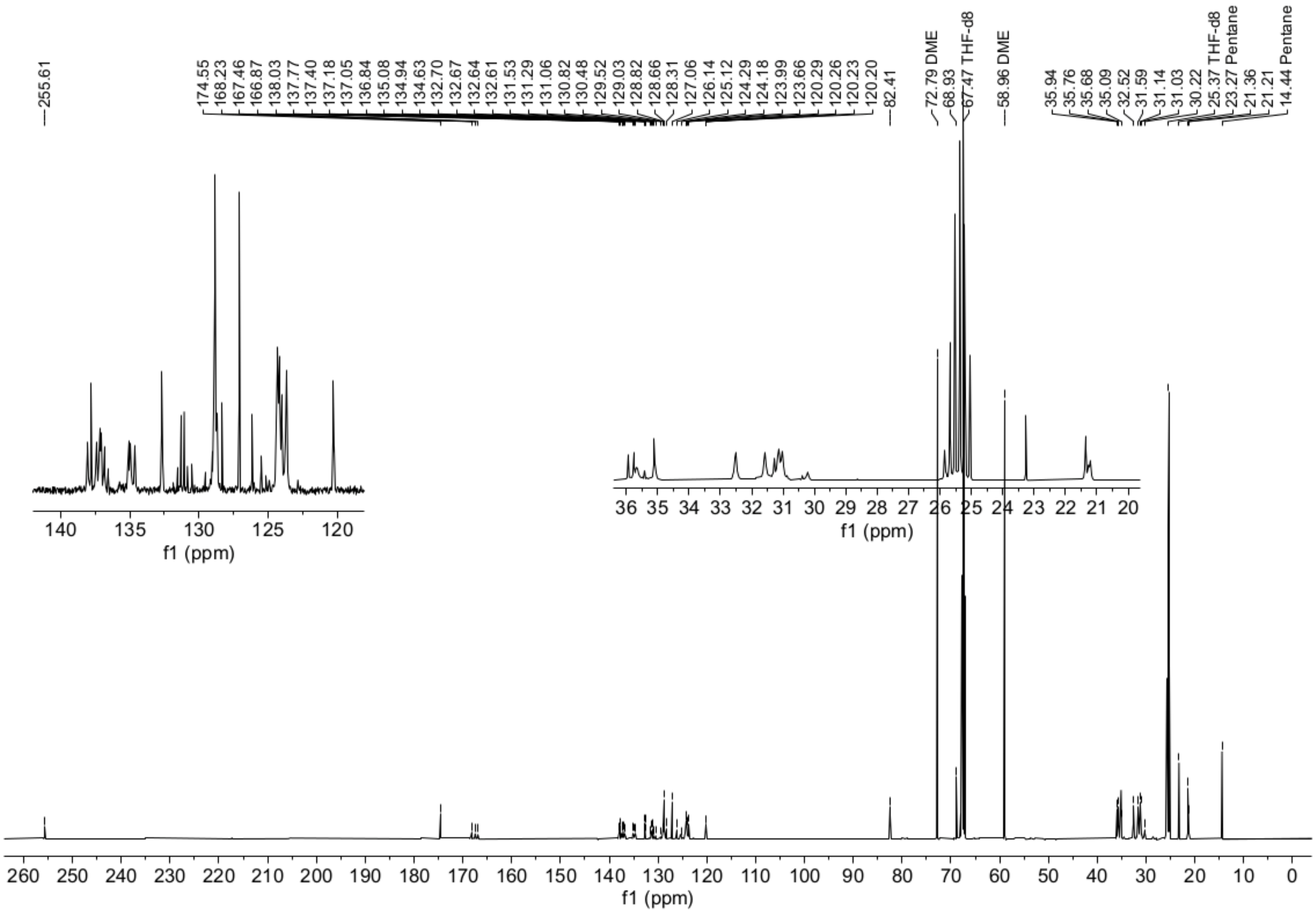

Supplementary Figure 15: $^{13}C\{^1H\}$ NMR (126 MHz, THF-d_8) spectrum of **3-DME.**

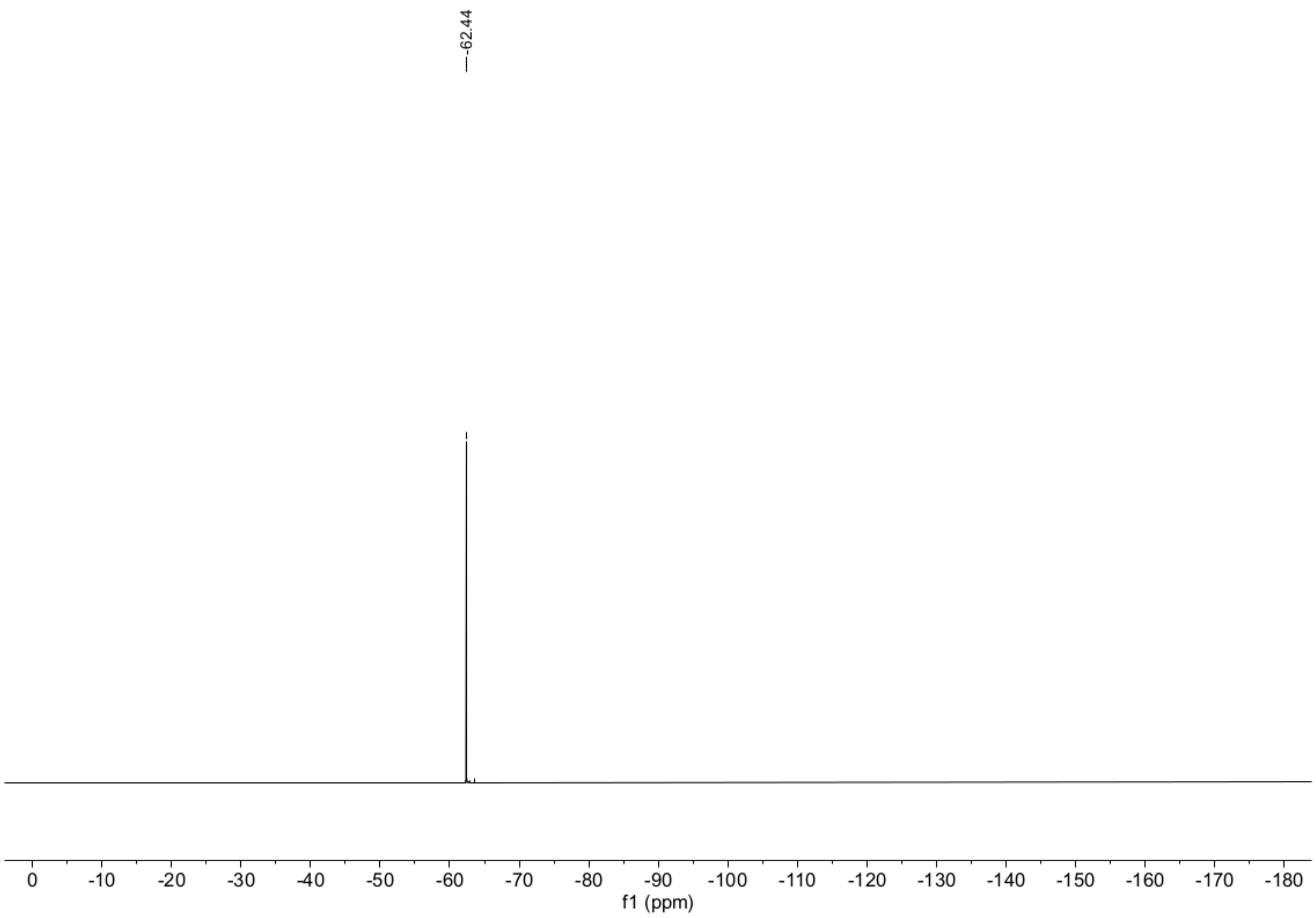

Supplementary Figure 16: ^{19}F NMR (470 MHz, THF-d_8) spectrum of **3-DME.**

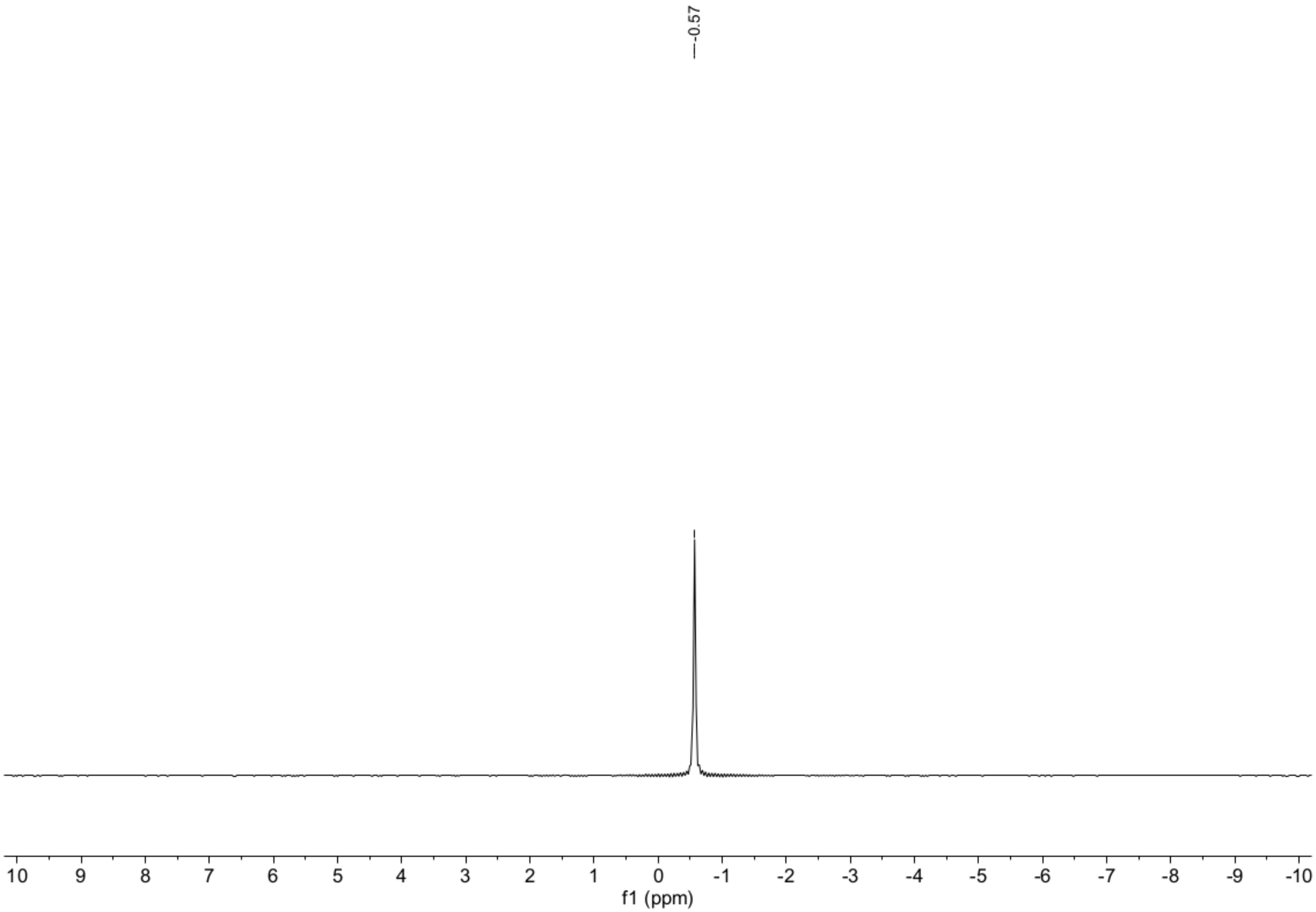

Supplementary Figure 17: ^{7}Li NMR (194 MHz, THF-d_8) spectrum of **3-DME.**

Electrochemical Measurements:

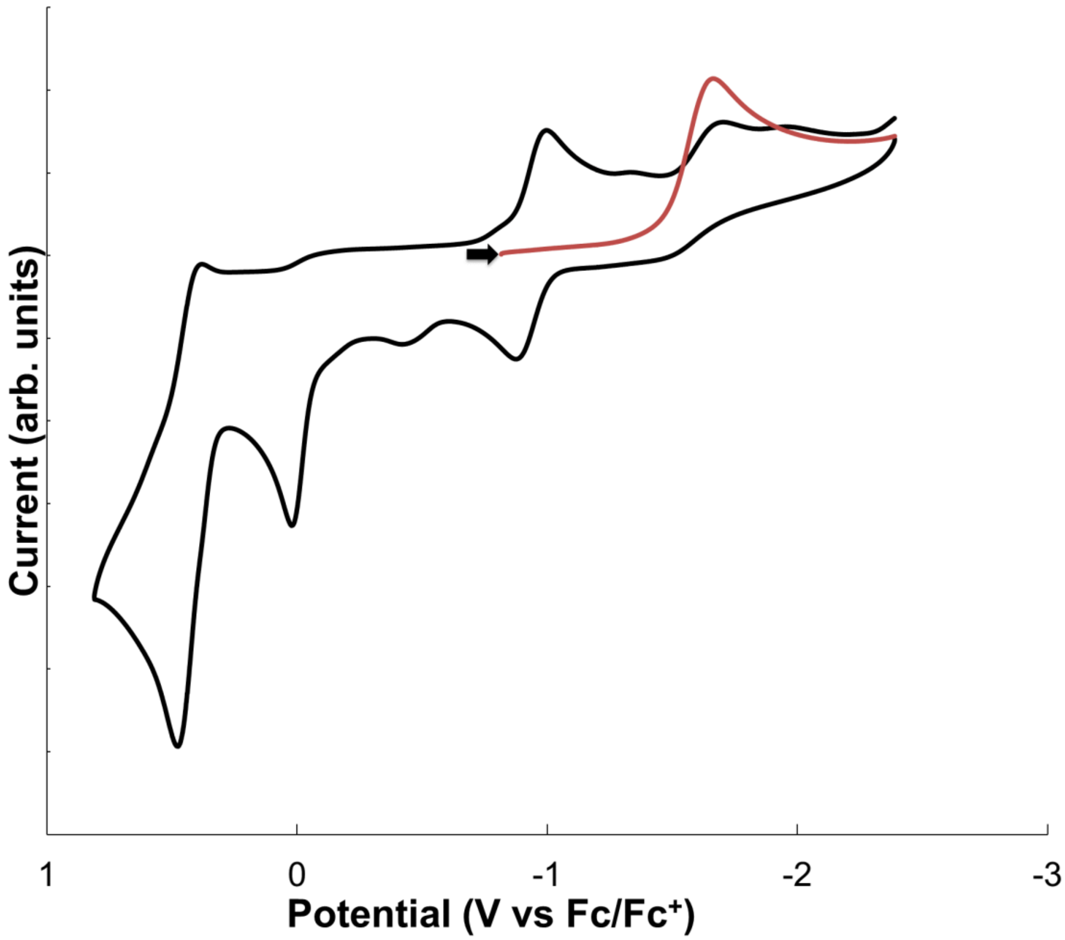

Supplementary Figure 18: Cyclic voltammogram of **3-THF** (1 mmol*L^{-1}) in THF using [^{n}NPr$_4$][BAr$^F{}_4$] (100 mmol*L^{-1}) as the supporting electrolyte. Scan Rate: 100 mV s^{-1}. OCP: −0.82 V vs Fc/Fc$^+$. Trace in red is the first scan, trace in black is representative of subsequent scans.

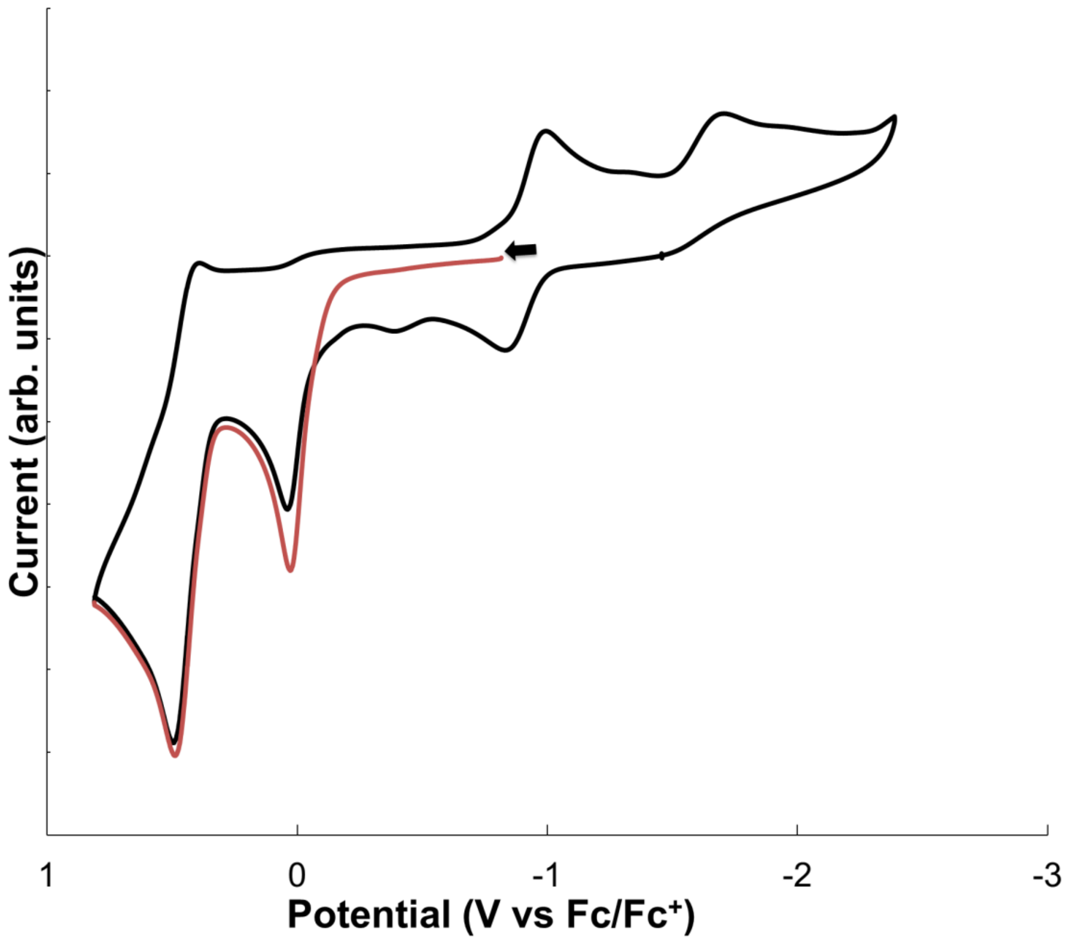

Supplementary Figure 19: Cyclic Voltammogram of **3-THF** (1 mmol*L^{-1}) in THF using [^{n}NPr$_4$][BAr$^F_4$] (100 mmol*L^{-1}) as the supporting electrolyte. Scan Rate: 100 mV s^{-1}. OCP: −0.82 V vs Fc/Fc$^+$. Trace in red is the first scan, trace in black is representative of subsequent scans.

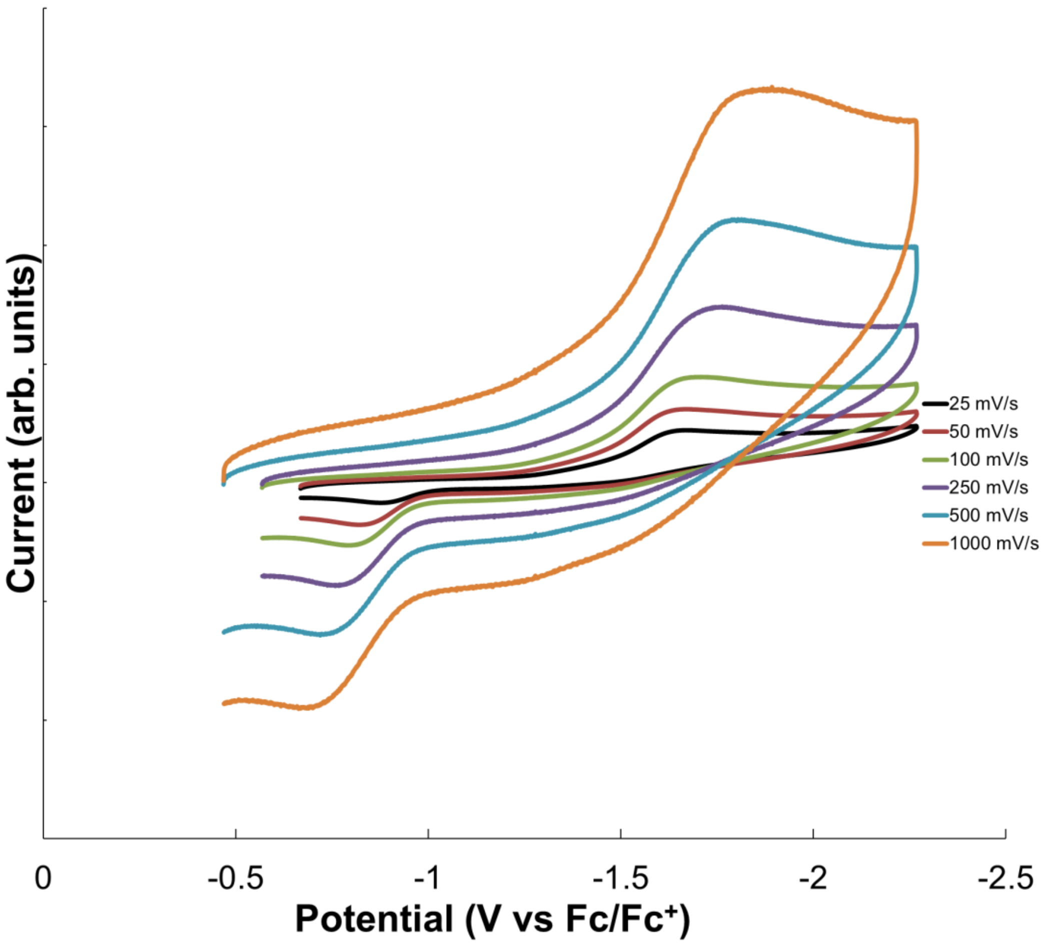

Supplementary Figure 20: Scan rate dependence of **3-THF** (1 mmol L^{-1}) recorded in THF using [$^{n}NPr_4$][BAr^F_4] (100 mmol L^{-1}) as the supporting electrolyte.

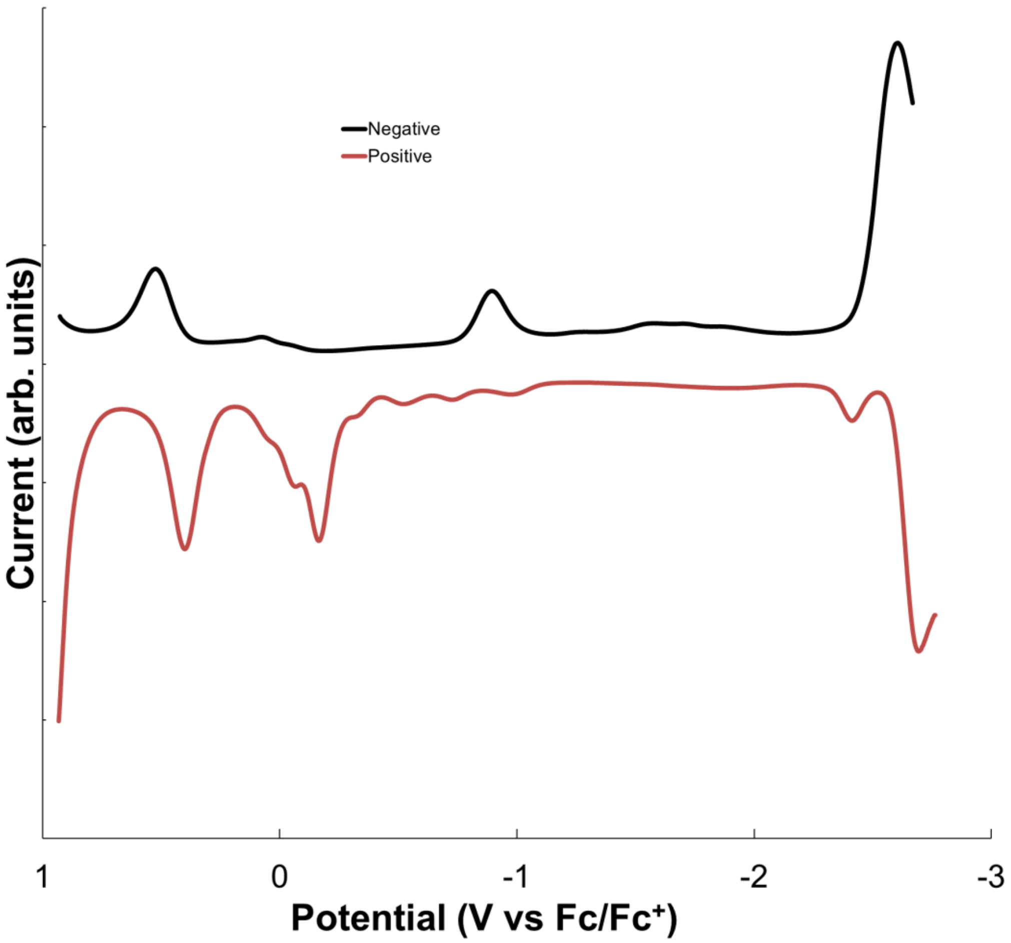

Supplementary Figure 21: Differential pulse voltammetry of **3-THF** (1 mmol L^{-1}) recorded in THF using [$^{n}NPr_4$][BAr^F_4] (100 mmol*L^{-1}) as the supporting electrolyte.

Electronic Spectra:

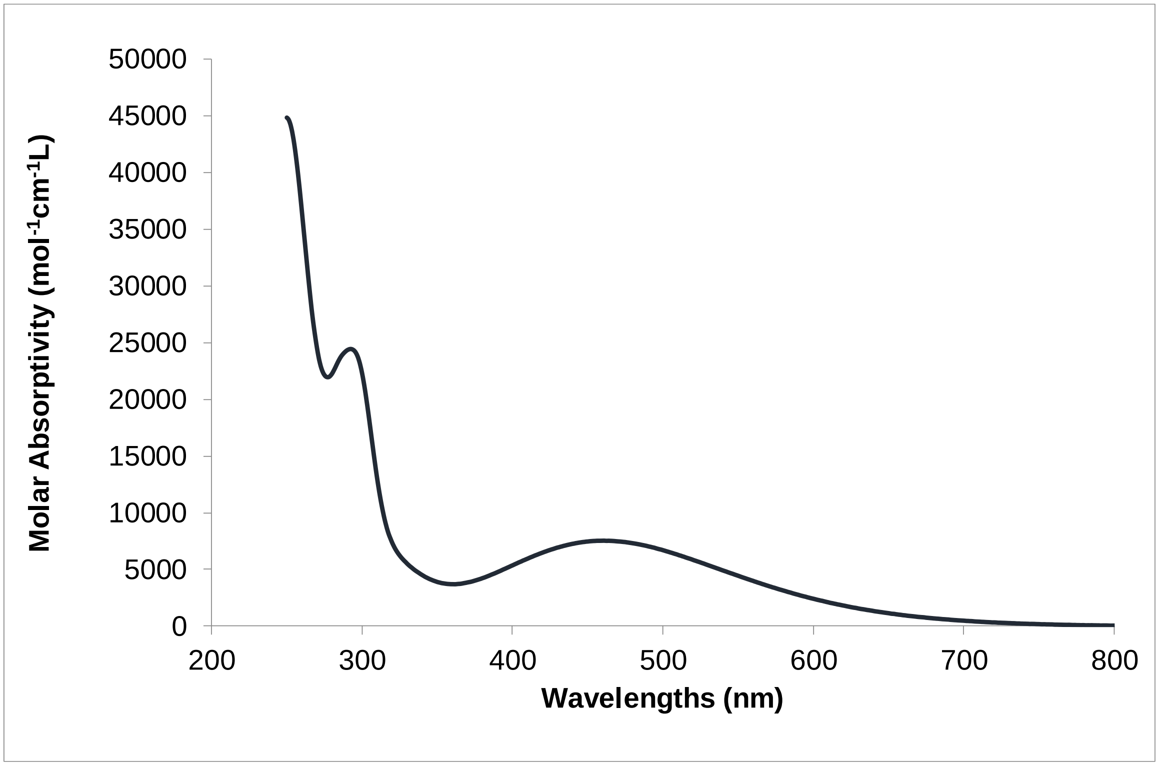

Supplementary Figure 22: UV-vis spectrum of **3-THF** recorded in THF.

X-ray crystal structures:

Collection Parameters:

Supplementary Table 1: SCXRD collection parameters

Compound	3-THF	3-DME
**Empirical formula**	$C_{81}H_{111}CeF_3LiNO_9$	$C_{75}H_{113}CeF_3LiNO_{11}$
**Formula weight**	1446.76	1408.72
**Temperature/K**	100	100
**Crystal system**	orthorhombic	orthorhombic
**Space group**	Pbca	Pbca
**a**	20.10380(8)Å	22.0007(2)Å
**b**	35.40366(14)Å	22.7743(3)Å
**c**	42.97760(17)Å	29.8804(3)Å
**Volume**	30589.2(2)Å^3	14971.6(3)Å^3
**Z**	16	8
**d_{calc}**	1.257 g/cm^3	1.250 g/cm^3
**F(000)**	12224.0	5968.0
**Crystal size, mm**	0.24 × 0.23 × 0.14	0.29 × 0.24 × 0.13
**2θ range for data collection**	4.992 – 149.006°	4.598 - 56.564°
**Reflections collected**	499971	299760
**Goodness-of-fit on F^2**	1.087	1.042
**Final R indexes [I>=2σ (I)]**	R_1 = 0.1043, wR_2 = 0.2942	R_1 = 0.0328, wR_2 = 0.0830

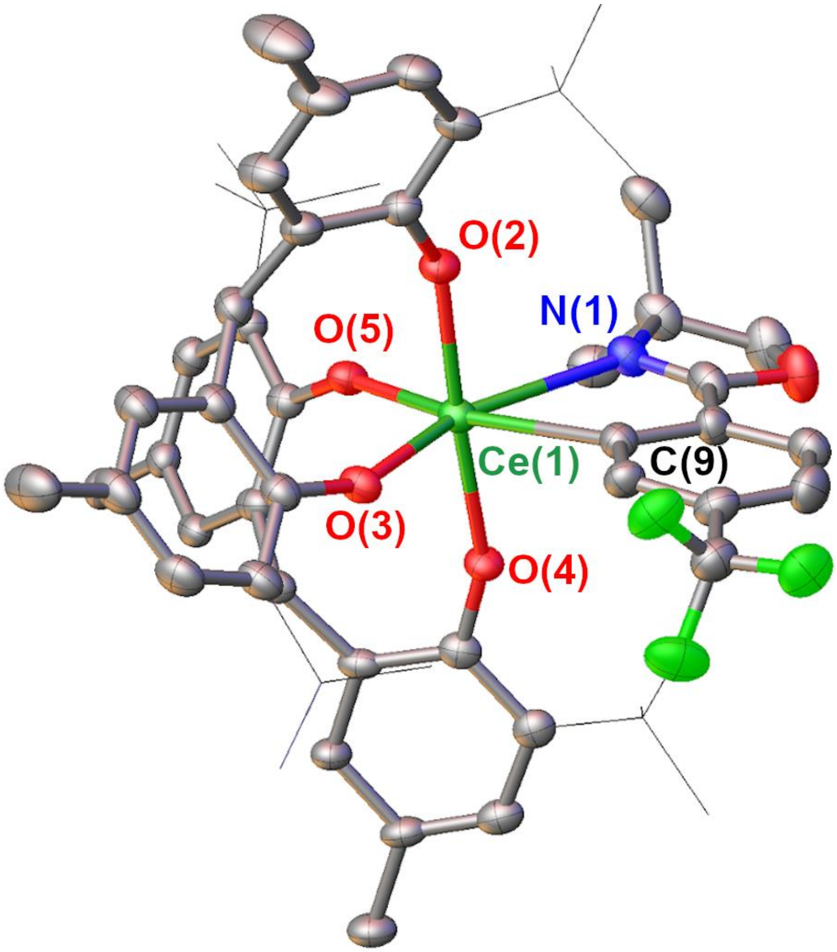

Supplementary Figure 23: Thermal ellipsoid plot of a portion of the asymmetric unit of **3-THF** showing one of the two unique complexes at the 30% probability level. *t*Bu groups are shown in wireframe and hydrogens are removed for clarity. Selected bond distances (Å): Ce(1)−C(9): 2.571(7), Ce(1)−N(1): 2.640(6), Ce(1)−O(2): 2.178(4), Ce(1)−O(3): 2.178(4), Ce(1)−O(4): 2.191(4) Ce(1)−O(5): 2.180(4). Selected bond angles (degrees): O(2)−Ce(1)−O(4): 176.77(17), O(2)−Ce(1)−O(5): 92.19(17), O(2)−Ce(1)−N(1): 91.09(17), O(2)−Ce(1)−C(9): 87.89(19), O(3)−Ce(1)−O(2): 90.61(17), O(3)−Ce(1)−O(4): 92.08(17), O(3)−Ce(1)−O5: 102.25(18), O(3)−Ce(1)−N(1): 157.71(18), O(3)−Ce(1)−C(9): 92.0(2), O(4)−−Ce(1)−N(1): 85.74(17), O(4)−Ce(1)−C(9): 90.23(19), O(5)−Ce(1)−O(4): 88.99(17), O(5)−Ce(1)−N(1): 99.88(17), O(5)−Ce(1)−C(9): 165.72(19), C(9)−Ce(1)−N(1): 65.85(19).

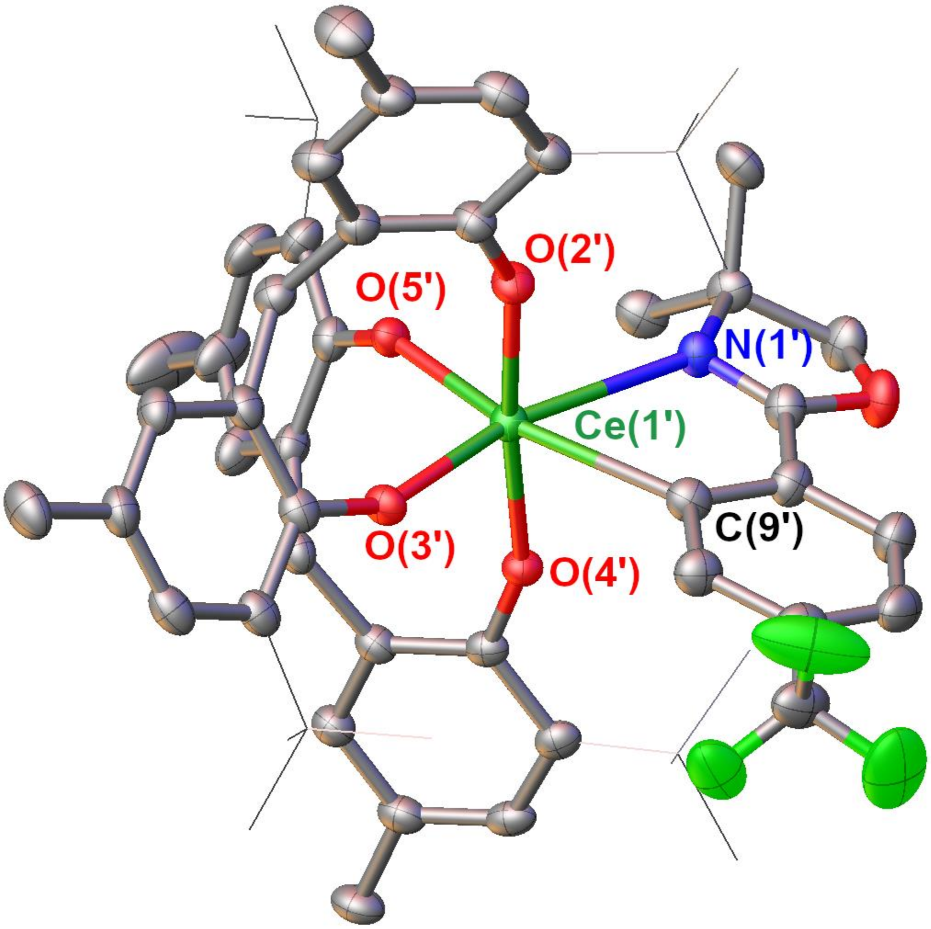

Supplementary Figure 24: Thermal ellipsoid plot of the second unique cerium complex in the asymmetric unit of **3-THF** at the 30% probability level. *t*Bu groups are shown in wireframe and hydrogens are removed for clarity. Selected bond distances (Å): Ce(1')−C(9'): 2.575(7), Ce(1')−N(1'): 2.623(5), Ce(1')−O(2'): 2.177(4), Ce(1')−O(3'): 2.181(4), Ce(1')−O(4'): 2.189(4), Ce(1')−O(5'): 2.202(4). Selected bond angles (degrees): O(2')−Ce(1')−O(3'): 87.90(16), O(2')−Ce(1')−O(4'): 173.87(16), O(2')−Ce(1')−O(5'): 94.57(16), O(2')−Ce(1')−N(1'): 92.03(17), O(2')−Ce(1')−C(9'): 87.92(19), O(3')−Ce(1')−O(4'): 93.62(16), O(3')−Ce(1')−O(5'): 98.49(16), O(3')−Ce(1')−N(1'): 163.86(17), O(3')−Ce(1')−C(9'): 97.69(19), O(4')−Ce(1')−O(5'): 91.08(15), O(4')−Ce(1')−N(1'): 84.87(16), O(4')−Ce(1')−C(9'): 86.00(18), O(5')−Ce(1')−N(1'): 97.61(16), O(5')−Ce(1')−C(9'): 163.71(18), C(9')−Ce(1')−N(1'): 66.19(19).

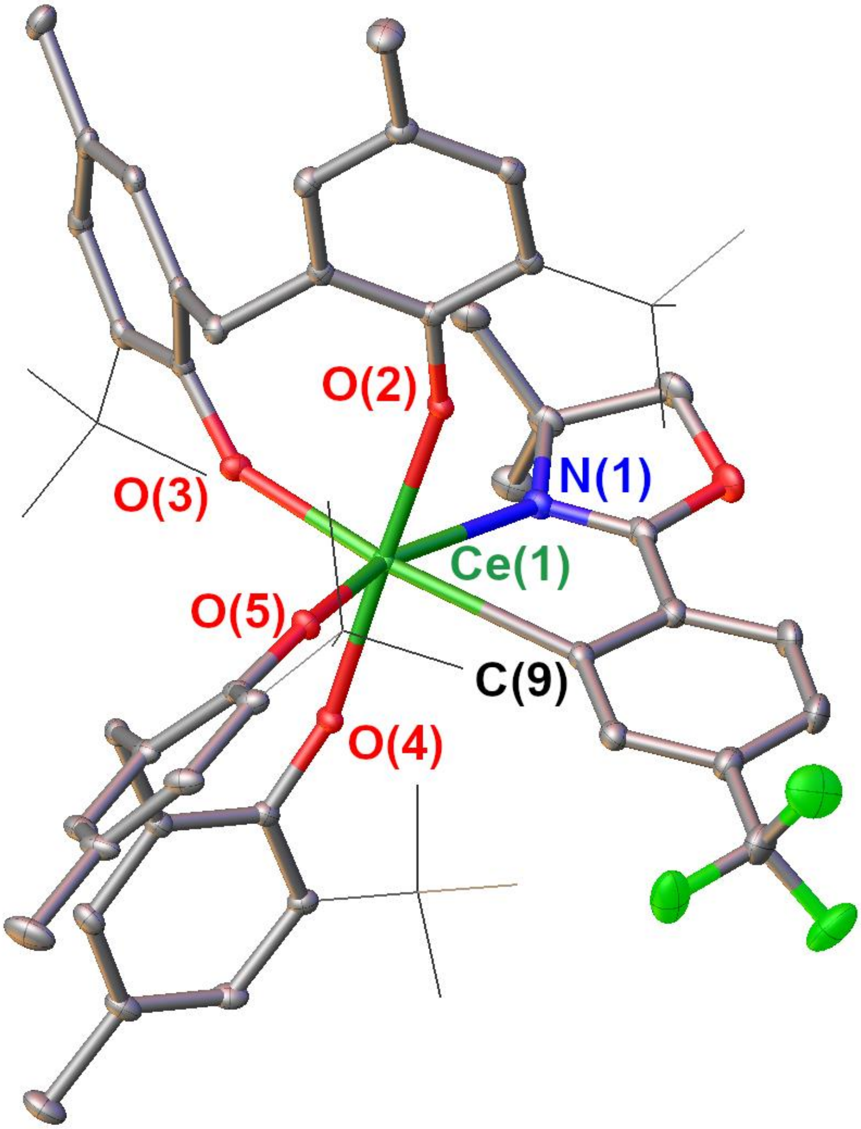

Supplementary Figure 25: Thermal ellipsoid plot of the cerium containing portion of the asymmetric unit of **3-DME** at the 30% probability level. *t*Bu groups are shown in wireframe and hydrogens are removed for clarity. Selected bond distances (Å): Ce(1)−C(9): 2.5806(19), Ce(1)−N(1): 2.6176(16), Ce(1)−O(2): 2.1750(12), Ce(1)−O(3): 2.2062(13), Ce(1)−O(4): 2.1640(12), Ce(1)−O(5): 2.1636(13). Selected bond angles (degrees): O(2)−Ce(1)−O(4): 91.16(5), O(2)−Ce(1)−O(5): 177.92(5), O(2)−Ce(1)−N(1): 83.80(5), O(2)−Ce(1)−C(9): 90.82(5), O(3)−Ce(1)−O(2): 90.19(5), O(3)−Ce(1)−O(4): 99.71(5), O(3)−Ce(1)−O5: 91.70(5), O(3)−Ce(1)−N(1): 98.67(5), O(3)−Ce(1)−C(9): 164.37(6), O(4)−Ce(1)−N(1): 160.95(5), O(4)−Ce(1)−C(9): 95.87(6), O(5)−Ce(1)−O(4): 87.67(5), O(5)−Ce(1)−N(1): 96.77(5), O(5)−Ce(1)−C(9): 87.59(5), C(9)−Ce(1)−N(1): 65.94(6).

Additional Computational Data:

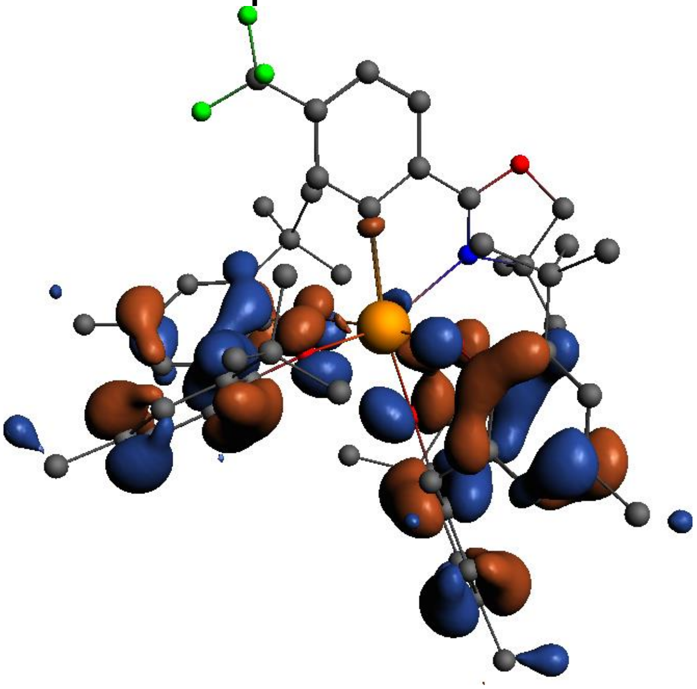

Supplementary Figure 26: The DFT/B3LYP frontier Kohn-Sham molecular orbitals of the HOMO-3 of **3**

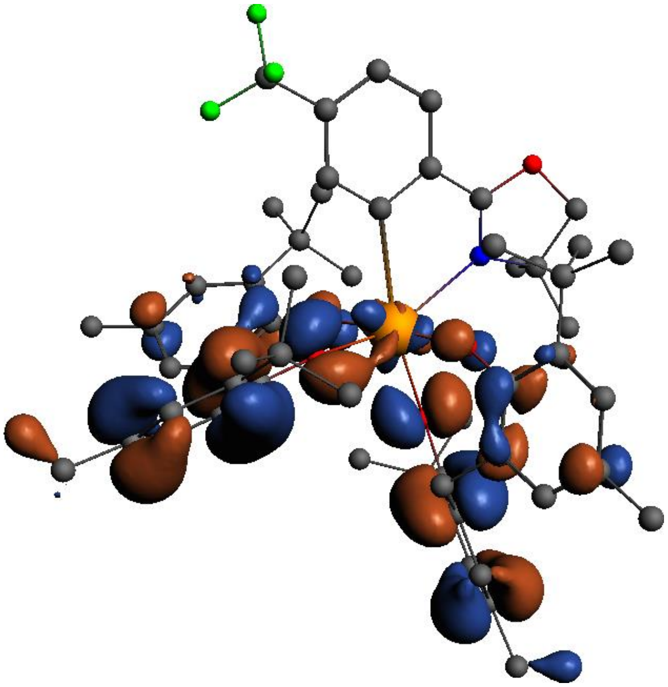

Supplementary Figure 27: The DFT/B3LYP frontier Kohn-Sham molecular orbitals of the HOMO-2 of **3**

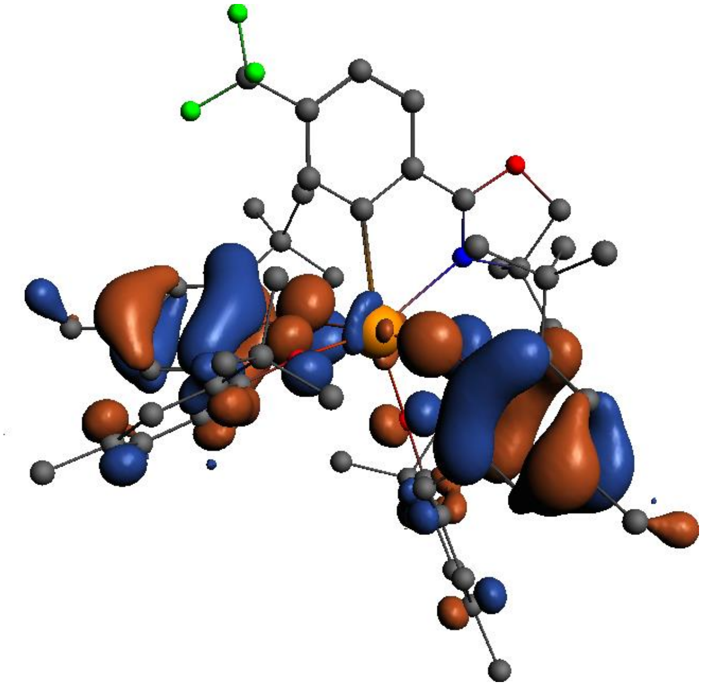

Supplementary Figure 28: The DFT/B3LYP frontier Kohn-Sham molecular orbitals of the HOMO-1 of **3**

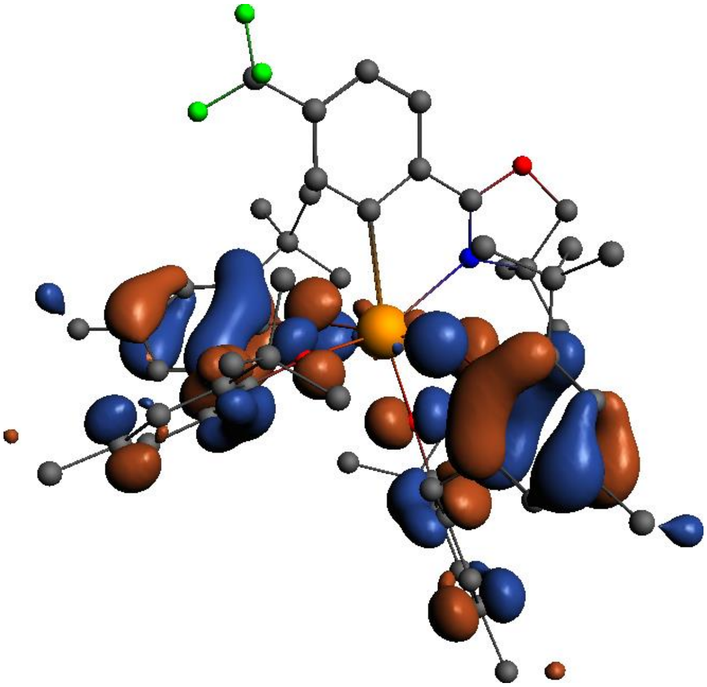

Supplementary Figure 29: The DFT/B3LYP frontier Kohn-Sham molecular orbitals of the HOMO of **3**

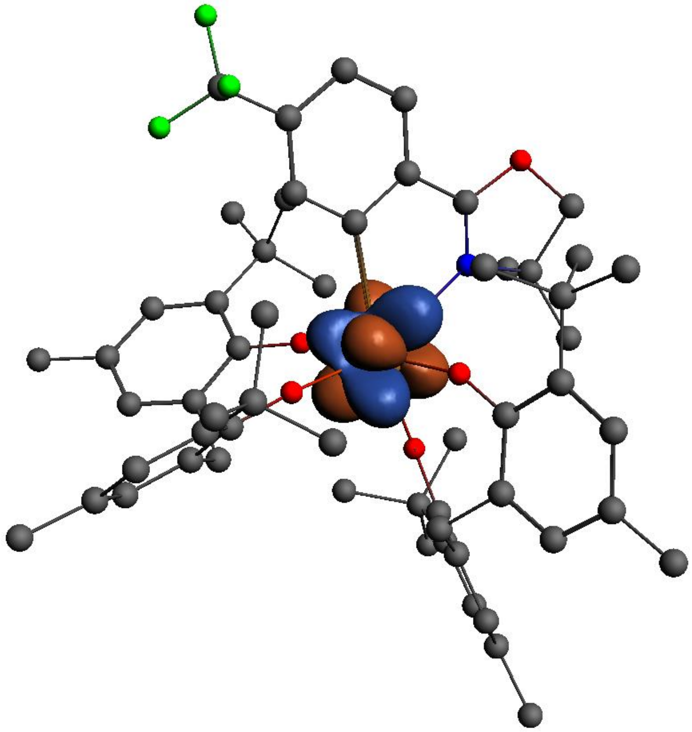

Supplementary Figure 30: The DFT/B3LYP frontier Kohn-Sham molecular orbitals of the LUMO of **3**

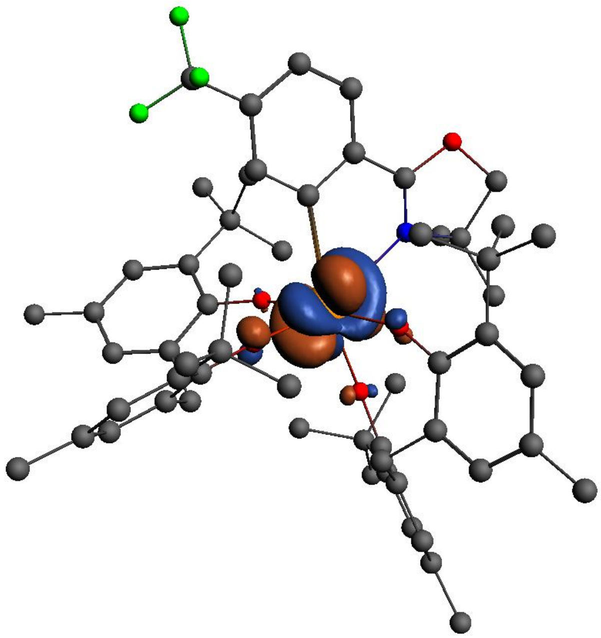

Supplementary Figure 31: The DFT/B3LYP frontier Kohn-Sham molecular orbitals of the LUMO+1 of **3**

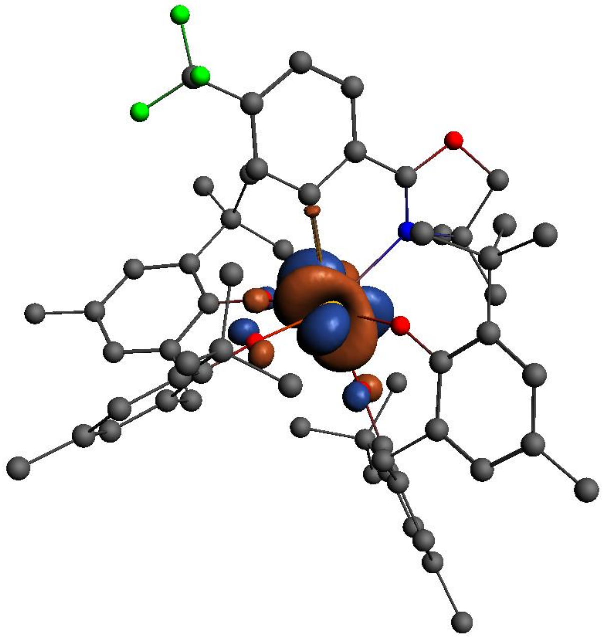

Supplementary Figure 32: The DFT/B3LYP frontier Kohn-Sham molecular orbitals of the LUMO+2 of **3**

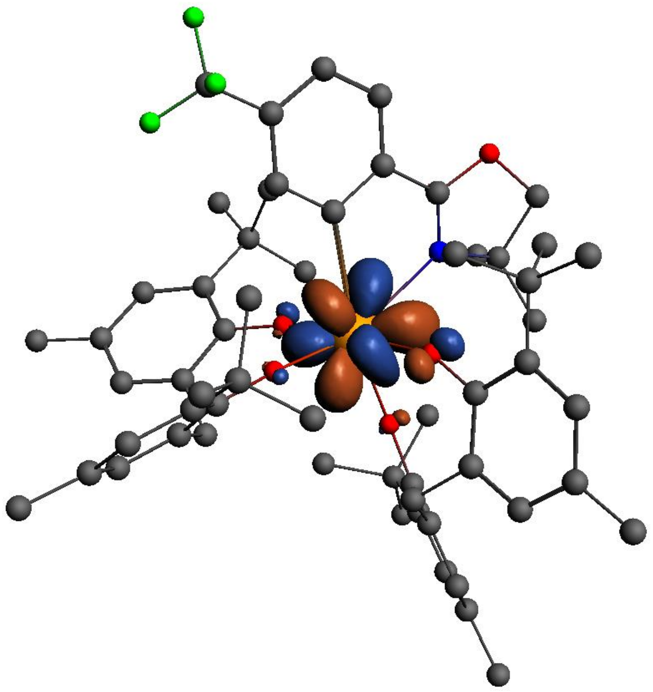

Supplementary Figure 33: The DFT/B3LYP frontier Kohn-Sham molecular orbitals of the LUMO+3 of **3**

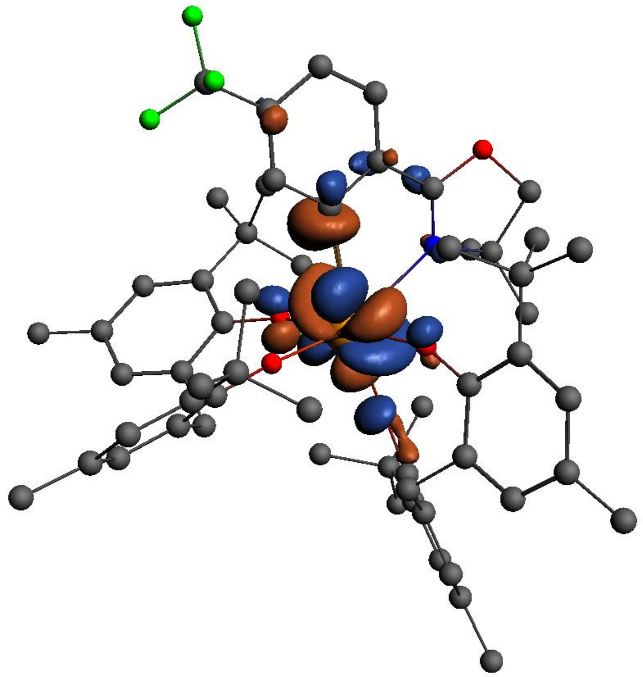

Supplementary Figure 34: The DFT/B3LYP frontier Kohn-Sham molecular orbitals of the LUMO+4 of **3**

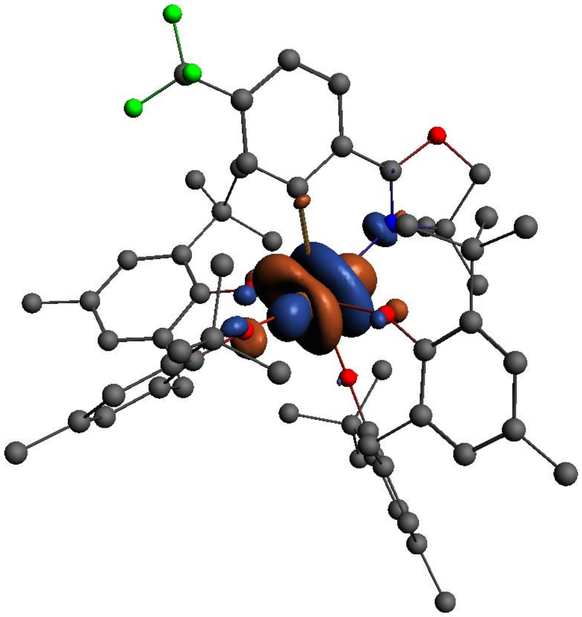

Supplementary Figure 35: The DFT/B3LYP frontier Kohn-Sham molecular orbitals of the LUMO+5 of **3**

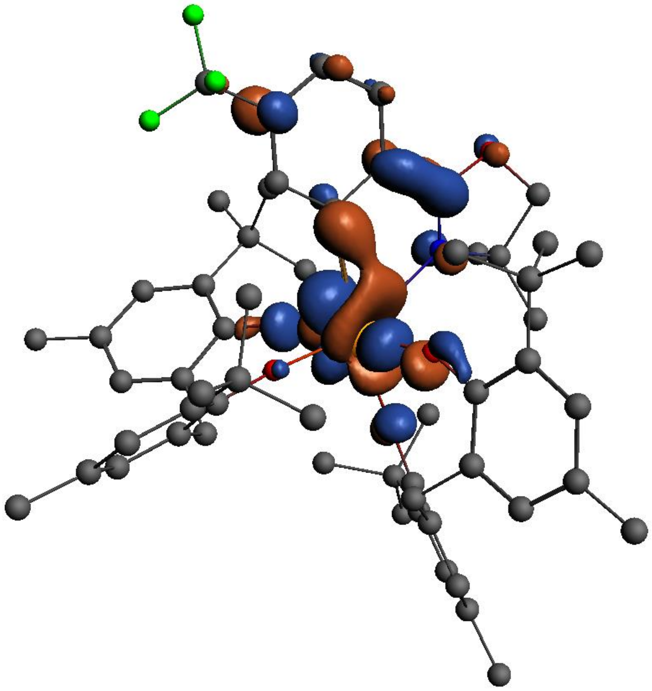

Supplementary Figure 36: The DFT/B3LYP frontier Kohn-Sham molecular orbitals of the LUMO+6 of **3**

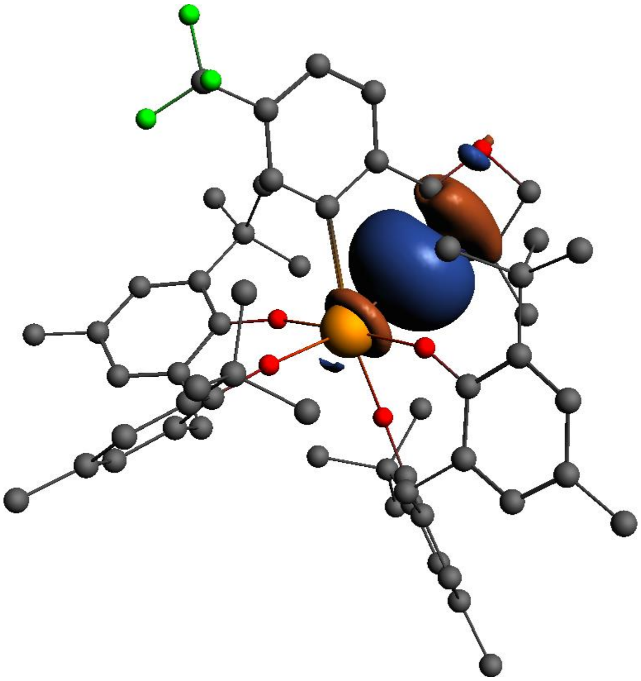

Supplementary Figure 37: The two-center two-electron bonding NLMO illustrating the chemical bond between the Ce center and the coordinating oxazoline N atom.

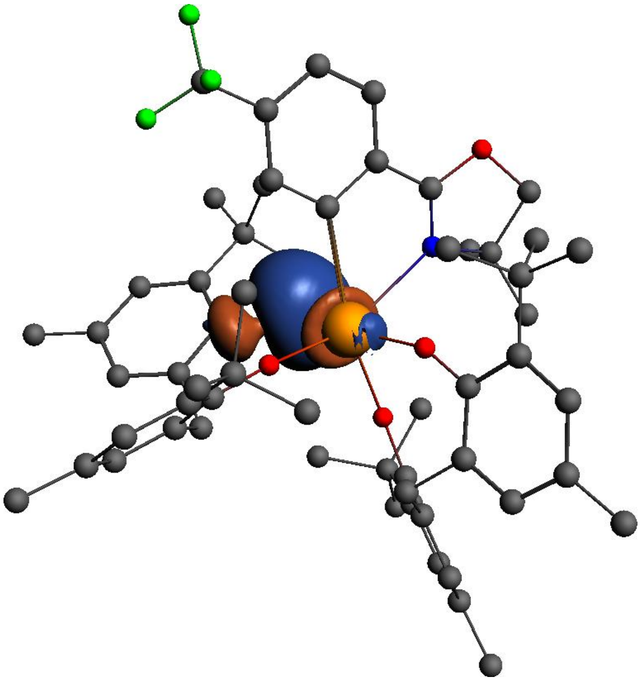

Supplementary Figure 38: The two-center two-electron bonding NLMO illustrating the σ chemical bond between the Ce center and the coordinating phenolate O atom.

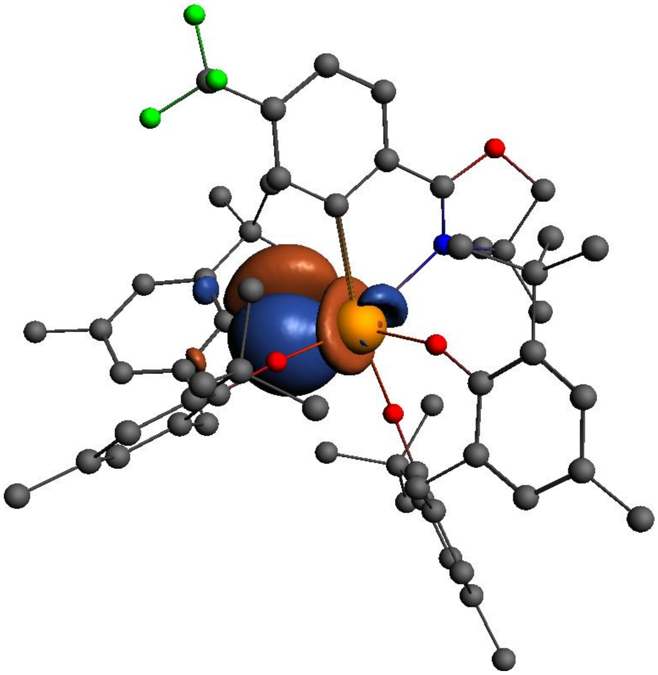

Supplementary Figure 39: The two-center two-electron bonding NLMO illustrating the π chemical bond between the Ce center and the coordinating phenolate O atom.

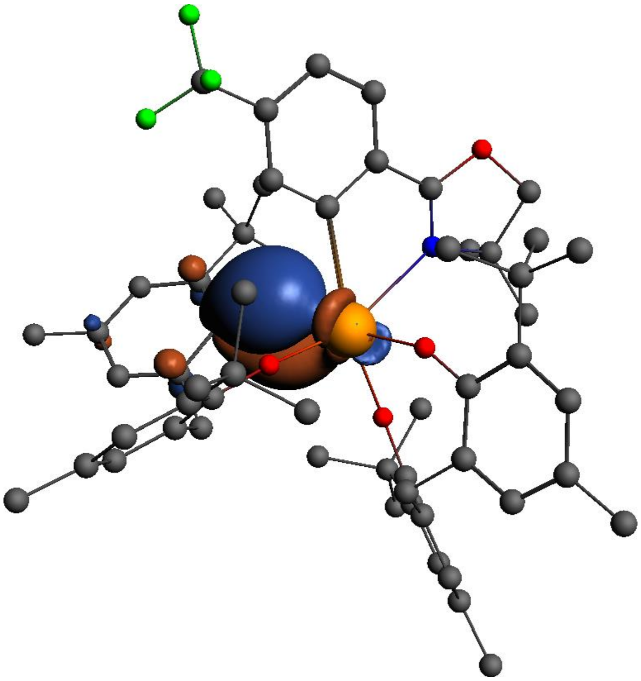

Supplementary Figure 40: The two-center two-electron bonding NLMO illustrating the π chemical bond between the Ce center and the coordinating phenolate O atom.

C_{aryl} NMR shift Calculations:

Supplementary Table 2: Calculated NMR chemical shielding (σ) and shift (δ) for the C_{aryl} atom center of **3**, using various approaches.

Approach	Relativity	σ	δ(ppm)	δ(ppm), expt.
B3LYP	SR	-44	230	256
	SO	-100	287	
PBE0	SR	-33	224	
	SO	-84	276	
PBEh-40[a]	SR	-26	219	
	SO	-65	**259**	
PBE	SR	-43	230	
	SO	-91	279	
KT2	SR	-21	213	
	SO	-71	**265**	
SAOP	SR	-28	216	
	SO	-53	**241**	

[a]PBE hybrid functional with 40% exact exchange.

Further QTAIM Data:

Supplementary Table 3: Further QTAIM bonding parameters

Bond/NLMO	$\rho^{\text{bcp a}}$	$\Delta\rho^{\text{bcp a}}$	$G^{bcp\ b}$	$V^{bcp\ b}$	$H^{bcp\ b}$	ε^{c}
σ(Ce–C)	0.062	0.080	0.041	–0.062	–0.021	0.08
σ(Ce–N)	0.041	0.126	0.035	–0.038	–0.003	0.14
σ(Ce–O_1)	0.089	0.355	0.110	–0.131	–0.021	0.04

[a]Density (ρ) and its Laplacian (Δρ) at the bond critical point (bcp);
[b]Kinetic (G), potential (V), and total (H) energy density at the bcp.
[c]Bond ellipticity.

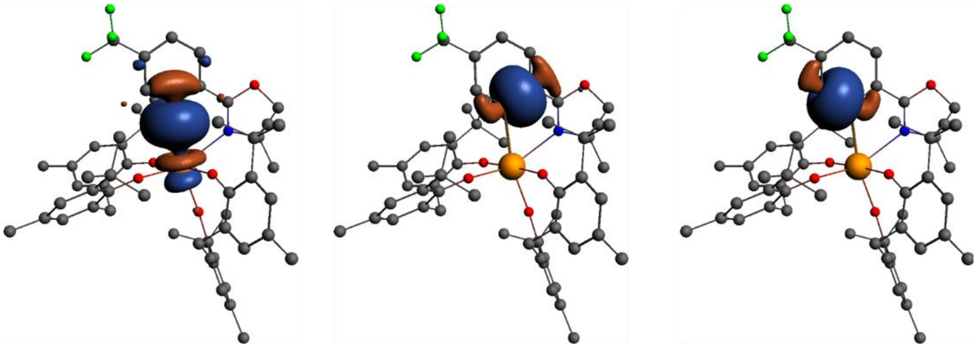

σ(Ce-C_{aryl}) 11%Ce(41%d; 53%f) | σ(C_{aryl}-C_1) | σ(C_{aryl}-C_2)

Supplementary Figure 41. NLMOs (±0.03 au isosurfaces), obtained with DFT/PBEh-40, that give the most important contributions to the Ce-bound C_{aryl} isotropic shielding (σ_{iso}) in Table 1. The Ce density weight in the σ(Ce-C_{aryl}) NLMO is 11%, with 41% and 53% contributions from the Ce 5d and 4f AOs respectively. These values are similar to those obtained with DFT/B3LYP reported in **Table 1**.

XYZ optimized coordinates for 3.
ADF
DFT/B3LYP(GD3BJ)/SR-ZORA, NumericalQuality(GOOD)
TZ2P(Ce, N, C, O, f) + TZP(H) + Small Frozen Core
COSMO(THF)
Bond Energy: –35.749068

Ce	0.00000000	-0.00000000	2.58048672
C	0.00000000	0.00000000	0.00000000
O	-2.14753936	-0.38507174	2.54378808
O	-0.41596673	2.13417762	2.64072474
O	2.14649434	0.39590108	2.75684559
O	0.01069072	-0.62225231	4.68974697
N	0.73307716	-2.28038686	1.48756578
C	1.24009454	-3.58053270	1.99885697
O	1.64294907	-3.26231827	-0.31060572
C	2.09601779	-4.07364544	0.81202713
H	3.15938699	-3.87991790	0.94845516
H	1.93516026	-5.11785999	0.55877039
C	0.96636865	-2.22485172	0.22640758
C	0.55106917	-1.12271347	-0.64071529
C	0.67628149	-1.22363527	-2.02947627
H	1.11738750	-2.10259154	-2.48061654
C	0.21839718	-0.19005175	-2.82937859
H	0.30336615	-0.25250753	-3.90510259
C	-0.36498722	0.92328192	-2.22592281
C	-0.46661320	1.01225957	-0.83853695
H	-0.92589550	1.89391593	-0.41099990
C	2.05577649	-3.37684576	3.27083439
H	1.43288012	-2.96299598	4.06203139
H	2.88034802	-2.68792214	3.09431382
H	2.46102784	-4.32992472	3.61525678
C	0.04695474	-4.51088877	2.22715409
H	-0.48921325	-4.67756884	1.29170960
H	-0.64805918	-4.08095120	2.94255254
H	0.39014398	-5.47414675	2.60959031
C	-0.82695336	2.06585839	-3.07893690
F	-1.86226730	2.74580900	-2.53374672
F	-1.22819225	1.67505750	-4.31516895
F	0.16087717	2.98472138	-3.28165481
C	-3.41350432	0.02246458	2.55177421
C	-4.41584457	-0.70395187	1.85280046
C	-5.71005434	-0.19089585	1.84766909
H	-6.48634802	-0.71978321	1.31230240
C	-6.06854609	0.97908194	2.51903150
C	-5.08424489	1.62863625	3.25079744
H	-5.34497515	2.51368179	3.82159840
C	-3.77002090	1.17142769	3.28933447
C	-2.77386981	1.88148591	4.18742523
H	-3.27666929	2.07207563	5.13849961
H	-1.94457283	1.21481204	4.41188341
C	-2.24221569	3.20607945	3.68410286
C	-2.92197369	4.37953251	3.99496105
H	-3.83898677	4.31597908	4.57129290
C	-2.44924071	5.62535464	3.60259078
C	-1.23456907	5.67071557	2.91928272
H	-0.85095751	6.64402527	2.64812662
C	-0.49532653	4.53387151	2.59386684
C	-1.03689732	3.26977739	2.95112509
C	-4.08798552	-2.03538485	1.16096656
C	-3.07484714	-1.81571985	0.02266011
H	-3.49203922	-1.15147536	-0.73772608

H	-2.83423769	-2.76971976	-0.45441953
H	-2.15545498	-1.37200658	0.38519818
C	-5.33206485	-2.69551928	0.54787024
H	-6.09559151	-2.90648978	1.29916503
H	-5.04128409	-3.64455911	0.09288340
H	-5.78016550	-2.07826146	-0.23309366
C	-3.51092131	-3.02234191	2.19654036
H	-2.61632828	-2.62441694	2.66423060
H	-3.26007263	-3.96892752	1.71147506
H	-4.24598578	-3.22375827	2.97912650
C	-7.47697343	1.51149357	2.44837381
H	-7.69045965	2.17655008	3.28683780
H	-8.21109285	0.70293444	2.46166661
H	-7.64305370	2.08210620	1.52901668
C	-3.21930927	6.88640895	3.90021917
H	-2.54940533	7.72907743	4.08242076
H	-3.85642060	6.76337662	4.77779534
H	-3.86850064	7.16448466	3.06372907
C	0.87777831	4.65844780	1.91453165
C	0.87587100	3.94309730	0.55290635
H	0.69624359	2.88156162	0.67164223
H	1.84284633	4.06975230	0.06058999
H	0.10439506	4.35641637	-0.10072941
C	1.95994236	4.03329777	2.81615467
H	1.98027134	4.52559568	3.79097322
H	2.94366909	4.14714218	2.35489236
H	1.78382160	2.97511478	2.96599175
C	1.27199798	6.12212951	1.66582974
H	0.57206008	6.62810435	0.99777525
H	2.25623936	6.14649297	1.19437697
H	1.33447687	6.69321034	2.59414202
C	3.27017169	0.70439036	3.39941490
C	4.52811630	0.65491244	2.73947971
C	5.67013054	0.92943167	3.48813630
H	6.63855381	0.88445976	3.00979501
C	5.63133630	1.28303674	4.83668170
C	4.38379275	1.40116420	5.43389672
H	4.31906038	1.73471793	6.46425576
C	3.20668561	1.12894384	4.74406307
C	1.88083023	1.39600105	5.42879121
H	2.00433005	2.31448719	6.00734139
H	1.12247873	1.61703226	4.68130605
C	1.35823090	0.34000641	6.38016646
C	1.79206620	0.34076026	7.70231827
H	2.54523339	1.06069501	8.00452757
C	1.27272534	-0.53608696	8.64602662
C	0.24407400	-1.38605590	8.24048757
H	-0.19734672	-2.02876873	8.98900825
C	-0.24643600	-1.42328920	6.93615830
C	0.37200566	-0.58364212	5.96970938
C	4.62533069	0.34205640	1.23887479
C	4.12301428	-1.08596579	0.97057351
H	4.76023716	-1.81517942	1.47653717
H	4.13889510	-1.30228171	-0.10040865
H	3.10879308	-1.20231016	1.33211253
C	3.78129631	1.35170181	0.43442717
H	2.73465358	1.31347369	0.71599214
H	3.85784056	1.13227405	-0.63343685
H	4.14407913	2.36788300	0.60204406
C	6.89993514	1.53298866	5.61116013
H	6.72056080	2.19298088	6.46174368
H	7.66756186	1.99000459	4.98330973

H	7.31831088	0.60132582	6.00562497
C	1.79138568	-0.56474423	10.06075871
H	2.57894030	-1.31540021	10.18212142
H	0.99933834	-0.81061748	10.77087871
H	2.21624466	0.39914299	10.34649588
C	-1.45230864	-2.30437156	6.57407615
C	-1.06083322	-3.34001163	5.50928721
H	-0.71989789	-2.84001472	4.61062486
H	-1.92115066	-3.96308140	5.25284773
H	-0.26154404	-3.99068673	5.87085507
C	-2.59331843	-1.42367998	6.02478054
H	-2.88304957	-0.66948161	6.75975327
H	-3.46772469	-2.04174404	5.80660675
H	-2.30008118	-0.92321652	5.10842563
C	-2.00005743	-3.07022018	7.78724020
H	-1.26025933	-3.75281574	8.20996860
H	-2.85826309	-3.66629996	7.47096661
H	-2.33655430	-2.39673641	8.57764814
C	6.06601589	0.43096676	0.71454288
H	6.72515742	-0.29067405	1.20079058
H	6.06757925	0.21170683	-0.35505735
H	6.48893988	1.42816609	0.85061747

References:

1 Adamo, C. & Barone, V. Toward chemical accuracy in the computation of NMR shieldings: the PBE0 model. *Chem. Phys. Lett.* **298**, 113-119, doi:10.1016/S0009-2614(98)01201-9 (1998).

2 Evans, W. J., Deming, T. J., Olofson, J. M. & Ziller, J. W. Synthetic and structural studies of a series of soluble cerium(IV) alkoxide and alkoxide nitrate complexes. *Inorg. Chem.* **28**, 4027-4034, doi:10.1021/ic00320a018 (1989).

3 Takaya, J., Sangu, K. & Iwasawa, N. Molybdenum(0)-Promoted Carbonylative Cyclization of o-Haloaryl- and β-Haloalkenylimine Derivatives by Oxidative Addition of a Carbon(sp_2)-Halogen Bond: Preparation of Two Types of γ-Lactams. *Angew. Chem. Int. Ed.* **48**, 7090-7093, doi:10.1002/anie.200902884 (2009).

4 SAINT (Bruker AXS, Inc, Madison, WI, USA, 2009).

5 SHELXTL (Bruker AXS, Inc, Madison, WI, USA, 2009).

6 SADABS (University of Gottingen, Germany, 2007).

7 TWINABS (University of Gottingen, Germany, 2008).

8 SHELXL 2014/7 (University of Gottingen, Germany, 2014).

9 Dolomanov, O. V., Bourhis, L. J., Gildea, R. J., Howard, J. A. K. & Puschmann, H. OLEX2: a complete structure solution, refinement and analysis program. *Journal of Applied Crystallography* **42**, 339-341, doi:10.1107/S0021889808042726 (2009).

10 Amsterdam Density Functional, Version 2017, SCM, Theoretical Chemistry (Vrije Universiteit, Amsterdam, The Netherlands, 2019).

11 Becke, A. D. Density-functional exchange-energy approximation with correct asymptotic behavior. *Phys. Rev. A.: At. Mol. Opt. Phys.* **38**, 3098-3100, doi:10.1103/PhysRevA.38.3098 (1988).

12 Lee, C., Yang, W. & Parr, R. G. Development of the Colle-Salvetti correlation-energy formula into a functional of the electron density. *Phys. Rev. B.: Condens. Matter* **37**, 785-789, doi:10.1103/PhysRevB.37.785 (1988).

13 Stephens, P. J., Devlin, F. J., Chabalowski, C. F. & Frisch, M. J. Ab Initio Calculation of Vibrational Absorption and Circular Dichroism Spectra Using Density Functional Force Fields. *J. Phys. Chem.* **98**, 11623-11627, doi:10.1021/j100096a001 (1994).

14 Lenthe, E. v., Baerends, E. J. & Snijders, J. G. Relativistic regular two‐component Hamiltonians. *J. Chem. Phys.* **99**, 4597-4610, doi:10.1063/1.466059 (1993).

15 Grimme, S., Antony, J., Ehrlich, S. & Krieg, H. A consistent and accurate ab initio parametrization of density functional dispersion correction (DFT-D) for the 94 elements H-Pu. *J. Chem. Phys.* **132**, 154104, doi:10.1063/1.3382344 (2010).

16 Grimme, S., Ehrlich, S. & Goerigk, L. Effect of the damping function in dispersion corrected density functional theory. *J. Comput. Chem.* **32**, 1456-1465, doi:10.1002/jcc.21759 (2011).

17 Glendening, E. D., Landis, C. R. & Weinhold, F. NBO 6.0: Natural bond orbital analysis program. *J. Comput. Chem.* **34**, 1429-1437, doi:10.1002/jcc.23266 (2013).
18 Bader, R. F. W. A quantum theory of molecular structure and its applications. *Chem. Rev.* **91**, 893-928, doi:10.1021/cr00005a013 (1991).
19 Perdew, J. P., Burke, K. & Ernzerhof, M. Generalized Gradient Approximation Made Simple. *Phys. Rev. Lett.* **77**, 3865-3868, doi:10.1103/PhysRevLett.77.3865 (1996).
20 Keal, T. W. & Tozer, D. J. The exchange-correlation potential in Kohn–Sham nuclear magnetic resonance shielding calculations. *J. Chem. Phys.* **119**, 3015-3024, doi:10.1063/1.1590634 (2003).
21 Gritsenko, O. V., Schipper, P. R. T. & Baerends, E. J. Approximation of the exchange-correlation Kohn–Sham potential with a statistical average of different orbital model potentials. *Chem. Phys. Lett.* **302**, 199-207, doi:10.1016/S0009-2614(99)00128-1 (1999).
22 Mahoney, B. D., Piro, N. A., Carroll, P. J. & Schelter, E. J. Synthesis, Electrochemistry, and Reactivity of Cerium(III/IV) Methylene-Bis-Phenolate Complexes. *Inorg. Chem.* **52**, 5970-5977, doi:10.1021/ic400202r (2013).
23 Jantzi, K. L., Guzei, I. A. & Reich, H. J. Solution and Solid-State Structures of Lithiated Phenyloxazolines. *Organometallics* **25**, 5390-5395, doi:10.1021/om060551j (2006).
